# Inhibitory effects of β-galactoside α2,6-sialyltransferase 1 on the Hippo pathway in breast cancer cells

**DOI:** 10.1016/j.jbc.2025.110266

**Published:** 2025-05-21

**Authors:** Qinglei Hang, Wenqian Li, Jingya Guo, Shiying Zuo, Yawen Yang, Can Wu, Wen Yong, Caimin Li, Jianguo Gu, Sicong Hou

**Affiliations:** 1 Key Laboratory of Jiangsu Province University for Nucleic Acid & Cell Fate Manipulation, Department of Clinical Medicine, Faculty of Medicine, Yangzhou University, Yangzhou, Jiangsu, China; 2 Jiangsu Provincial Innovation and Practice Base for Postdoctors, Suining People's Hospital, Affiliated Hospital of Xuzhou Medical University, Suining, Jiangsu, China; 3 Division of Regulatory Glycobiology, Institute of Molecular Biomembrane and Glycobiology, Tohoku Medical and Pharmaceutical University, Sendai, Miyagi, Japan; 4 Department of Gastroenterology, The Affiliated Hospital of Yangzhou University, Yangzhou University, Yangzhou, Jiangsu, China

**Keywords:** Hippo, *N*-linked glycosylation, glycosyltransferase, sialyltransferase, ST6GAL1

## Abstract

The Hippo signaling pathway is crucial in various pathological functions, such as tumor development. Yes-associated protein (YAP), a well-known downstream effector of the Hippo pathway, has been intensively studied; emerging evidence suggests that multiple cell membrane receptors can regulate the Hippo pathway. However, the mechanistic roles of these upstream pathways remain largely unknown. Here, we identified the β-galactoside α2,6-sialyltransferase 1 (ST6GAL1) catalyzed α2,6-sialylation as a pivotal upstream modulator of Hippo pathway by a glycosyltransferases overexpression sublibrary screening. Depletion of ST6GAL1 results in increased phosphorylation of large tumor suppressor kinase 1 and YAP, which induces YAP's nuclear localization, transcriptional activity, and multiple biological functions in breast cancer cells, including cell adhesion, spreading, growth, migration, and metastasis. These phenotypes were majorly due to the altered signal transduction of cell surface receptors, as deletion of ST6GAL1 exhibited attenuated G protein–coupled receptor, epidermal growth factor receptor, and integrins response and suppression of dephosphorylation of YAP. Mechanistically, these representative membrane receptors are α2,6-sialylated proteins, and their α2,6-sialylation could be inhibited by β-galactoside α2,3-sialyltransferase 4 *via* substrate competition. In addition, the α2,6-sialylation is essential for integrin β1–epidermal growth factor receptor/LPAR4 complex formation. Altogether, our findings demonstrate ST6GAL1 is an upstream negative regulator of the Hippo pathway in breast cancer cells, providing a new insight into the regulation between *N*-glycosylation and Hippo signaling.

The Hippo signaling pathway, first discovered in *Drosophila*, is a highly evolutionarily conserved pathway that restricts organ size and tumorigenesis (1). Accumulating evidence indicates that disruption of the Hippo pathway plays a vital role in the initiation and progression of a broad range of human cancers *via* a kinase cascade that finally enhances the activity of yes-associated protein (YAP) (2, 3, 4). Phosphorylation of YAP by the core components of the Hippo pathway, including the mammalian sterile 20-like kinases (MSTs) and large tumor suppressor kinases (LATSs), results in YAP cytoplasmic sequestration and ubiquitination-dependent proteasomal degradation (5). In contrast, inactivated Hippo signaling is an important oncogenic event; YAP nuclear accumulation and its association with TEAD family members have been demonstrated to promote the transcriptional activities that regulate the target genes involved in cancer cell proliferation or metastasis (6, 7). Previous studies have shown the core kinase cascade was modulated by large quantities of membrane proteins such as integrin, E-cadherin, G protein–coupled receptor (GPCR), and receptor tyrosine kinases (RTKs) (8, 9, 10), while the molecular mechanisms by which the Hippo pathway upstream membrane proteins in response to malignant behavior are still not clear.

As carriers of activities and functional performers in living organisms, proteins are often regulated by posttranslational modifications (PTMs) (11). Glycosylation is one of the most frequent posttranslational modifications, which could alter the structure and function of a protein, playing an essential role in cellular signal transduction, cellular interactions, and maintenance of cellular function (12). Almost all membrane proteins (like epidermal growth factor receptor (EGFR), integrins, TGF-β receptors, and PD-L1) can be modified by *N*-linked glycosylation, subsequently regulating the biological role *via* different tumor signals (13, 14, 15, 16, 17). *N*-linked glycosylation is one of the most widely and complex types of glycosylation modification, with glycan processing in the Golgi (Fig. 1*A*) through various glycosyltransferases (GTs).

**Figure 1:**
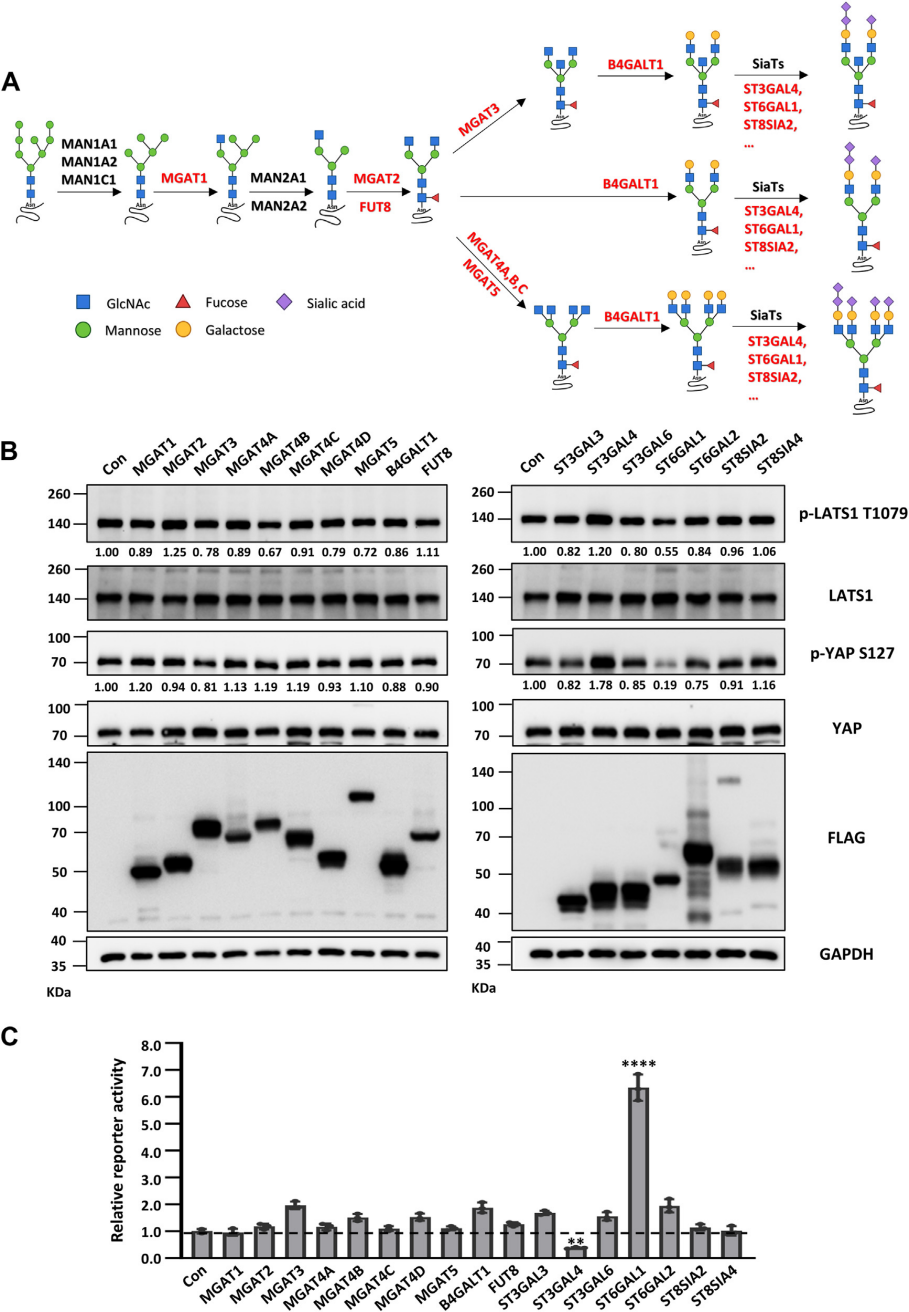
**Sialylation is correlated with the Hippo pathway.***A,* the sequential biosynthesis of typical *N*-linked glycoproteins within the Golgi in mammalian cells, the glycosyltransferases (GTs) are highlighted in *red*. *B,* cell lysates from GT overexpressed (GT-OE) HEK293T cells were immunoblotted by anti-p-YAP S127, anti-YAP, anti-p-LATS1 T1079, anti-LATS1, anti-FLAG, and anti-GAPDH antibodies. Quantification results were normalized to LATS1 or YAP. *C,* the luciferase activities in GTs-OE HEK293T cells cotransfected with 8 × GTIIC luciferase and TK-Renilla luciferase reporters were measured using a Dual-Luciferase Reporter Assay System. Error bars are mean ± SD (*n* = 3 biological replicates, ∗∗, *p* < 0.01, ∗∗∗∗, *p* < 0.0001 is determined by one-way ANOVA with Dunnet's test). LATS, large tumor suppressor kinase; YAP, yes-associated protein.

These unique biosynthetic pathways result in the generation of a large number of diverse and complex glycoprotein glycoforms, which enrich their functions. In detail, the TGF-β receptor might be modified by fucosyltransferase 8 (FUT8) to facilitate TGF-β binding and enhance downstream signaling, promoting the migratory and invasive capabilities in breast cancer cells, potentially leading to distal lung metastasis (18). Besides, increased bisecting GlcNAc residues on integrin β4 *via N*-acetylglucosaminyltransferase III (GnT-III) may contribute to drug resistance by suppressing caspase-3–mediated apoptosis in breast cancer (19). Moreover, α2,6-sialylation of EGFR can promote receptor dimer formation and activation, subsequently delivering downstream signaling *via* AKT and NF-κB, fostering resistance to radiotherapy and targeted therapies (17). However, the GTs that influence upstream receptors of the Hippo pathway are currently unknown.

To resolve these issues using a GTs overexpression sublibrary screening, we first identified ST6GAL1 as a key GT for Hippo signaling. We demonstrated that α2,6-sialylation catalyzed by ST6GAL1, but not ST3GAL4-mediated α2,3-sialylation, promotes breast cancer cell proliferation, migration, adhesion, spreading, and metastasis *via* inhibition of the Hippo pathway. Furthermore, we identified ST6GAL1-catalyzed α2,6-sialylation on several upstream cell membrane receptors of Hippo signaling, including LPAR4, EGFR, and integrin α5β1, which was essential for integrin β1–EGFR/LPAR4 complex formation and their signal transduction. These findings shed light on the *N*-glycosylation–mediated regulation of Hippo signaling.

## Results

### Sialylation, especially α2,6-sialylation, is negatively correlated with the Hippo signaling

The growing body of evidences suggest that the *N*-GTs have been reported to correlate with cancer malignancy (*e.g.*, progression and metastasis) by affecting the structures of attached glycans on cell membrane receptors, which are also reported as the upstream regulators of the Hippo pathway, we hypothesized a potential role of GTs, especially those involved in *N*-glycoprotein biosynthesis in Golgi (Fig. 1*A*), in the regulation of Hippo pathway. To test this idea, we performed a screening by stably overexpressing several key GTs, including MGAT1, MGAT2, MGAT3, MGAT4A, MGAT4B, MGAT4C, MGAT4D, MGAT5, B4GALT1, FUT8, ST3GAL3, ST3GAL4, β-galactoside α2,3-sialyltransferase 6 (ST3GAL6), ST6GAL1, ST6GAL2, ST8SIA2, and ST8SIA4, in HEK293T cells to investigate the role of *N*-glycosylation in Hippo signaling. As shown in Figure 1*B*, the phosphorylation of LATS1 on Thr1079 and YAP on Ser127 were significantly regulated after ST3GAL4 or ST6GAL1 overexpression. However, other candidate GTs exhibit little to no effect on the phosphorylation of LATS1(T1079) and YAP(S127) (Fig. 1*B*). Consistently, ST3GAL4- or ST6GAL1-overexpressed (OE) cells exhibited lower or higher activity of a YAP-TEAD luciferase reporter than control (Con) cells (Fig. 1*C*). These data indicated that sialylation, especially ST3GAL4- and ST6GAL1-mediated α2,3-sialylation and α2,6-sialylation, significantly regulated the phosphorylation of LATS1(T1079) and YAP(S127), implying play a key role in Hippo signaling.

**Figure 2:**
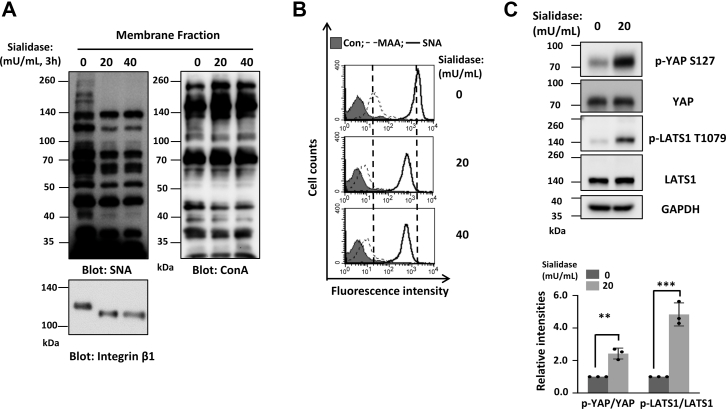
**Desialylation results in the activation of the Hippo pathway.***A,* MDA-MB-231 cells were pretreated with different doses of sialidase for 3 h; then, the cell membrane fractions were immunoblotted with SNA (recognizing α2,6-sialylated proteins) and ConA (an α-mannose/α-glucose-binding lectin) lectins or blotted with anti-integrin β1 antibody. *B,* to further determine the change of sialylation on the cell surface after sialidase treatment, the indicated cells were incubated with biotin-conjugated MAA (recognizing 2,3-sialylated proteins, *dotted line*), biotin-conjugated SNA (*bold line*), or without (*gray shadow*) lectin, followed by incubation with appropriate Alexa Flour 647 conjugate and subjected to flow cytometry. *C,* MDA-MB-231 cells were treated as described in (*A*), and then the cell lysates were immunoblotted with anti-p-YAP S127, anti-YAP, anti-p-LATS1 T1079, anti-LATS1, and anti-GAPDH antibodies. The relative ratios (phospho-YAP and phospho-LATS1 *versus* YAP and LATS1, respectively) are presented as the mean ± SD (*n* = 3 biological replicates, ∗∗, *p* < 0.01, ∗∗∗, *p* < 0.001 is determined by two-tail unpaired *t* test). SNA, *Sambucus nigra;* MAA, *Maackia amurensis* agglutinin; ConA, Concanavalin A; LATS, large tumor suppressor kinase; YAP, yes-associated protein.

To further confirm the effect of sialylation in regulating the Hippo signaling, we treated MDA-MB-231 cells with sialidase to remove sialic acid on the cell surface. As shown in Figure 2*A*, the reactivity of *Sambucus nigra* (SNA) lectin, which specifically recognizes α2,6-sialylation, was dramatically decreased upon sialidase treatment in cell membrane fractions and the mobility on SDS-PAGE of integrin β1 in the untreated group was slower than that of the sialidase-treated group. In addition, the flow cytometry analysis showed that the reactivity against SNA and *Maackia amurensis* agglutinin (MAA) (specifically recognize α2,3-sialylation) lectin was significantly decreased upon sialidase treatment (Fig. 2*B*). These results indicate the successful inhibition of sialylation in this cell line. Interestingly, treatment with sialidase resulted in stimulation of phosphorylation of LATS1(T1079) and YAP(S127) (Fig. 2*C*). Taken together, these results demonstrate that sialylation inhibit the phosphorylation of LATS1(T1079) and YAP(S127), implying that it suppresses the Hippo pathway in breast cancer cells.

### Knockout of the ST6GAL1 leads to YAP inactivation in breast cancer cells

**Figure 3:**
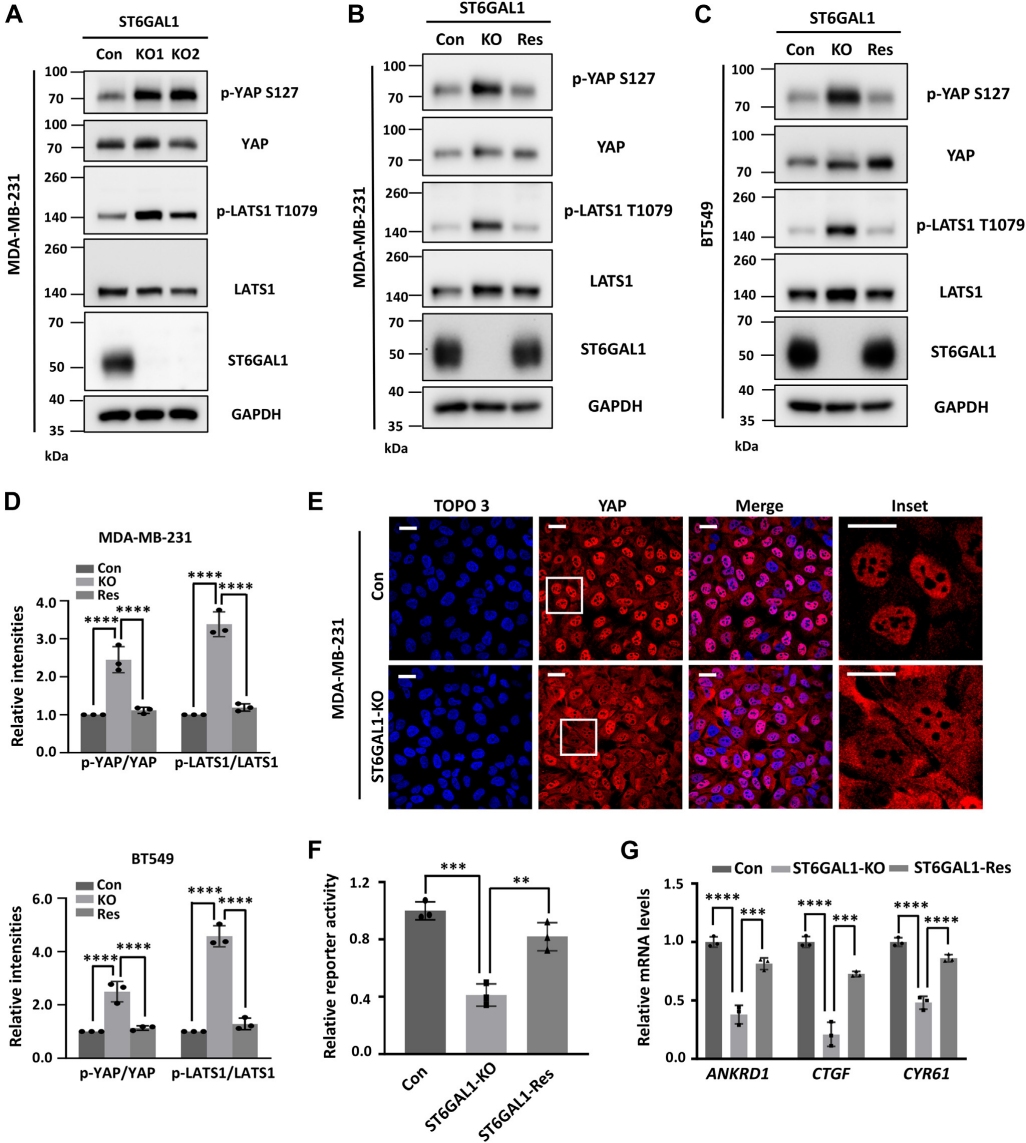
**Knockout of ST6GAL1 leads to phosphorylation and functional inactivation of YAP in breast cancer cells.***A–C,* cell lysates from the control (Con)-, ST6GAL1-KO1 (KO1), and ST6GAL1-KO2 (KO2)- MDA-MB-231 cells (*A*) and cell lysates from the control (Con)-, ST6GAL1-KO2 (KO)- and KO-ST6GAL1-rescue (Res)- MDA-MB-231 (*B*) and BT549 (*C*) cells were immunoblotted by anti-p-YAP S127, anti-YAP, anti-p-LATS1 T1079, anti-LATS1, anti-ST6GAL1, and anti-GAPDH antibodies. *D,* the relative ratios (phospho-YAP and phospho-LATS1 *versus* YAP and LATS1, respectively) are presented as the mean ± SD (*n* = 3 biological replicates, ∗∗∗∗, *p* < 0.0001 is determined by one-way ANOVA with Tukey's *post hoc* test). *E,* the expression and localization of YAP in Con- and ST6GAL1-KO- MDA-MB-231 cells. Con and KO cells were cultured on coverslips, and cells were fixed, permeabilized, and then visualized with YAP. Nuclei were stained with TO-PRO-3. The scale bar represents 20 μm. *F,* the luciferase activity in Con-, ST6GAL1-KO-, and ST6GAL1-Res- MDA-MB-231 cells. Cells were cotransfected with 8 × GTIIC luciferase and TK-Renilla luciferase reporters; the reporter activities were measured using a Dual-Luciferase Reporter Assay System. *G,* the mRNA expression of *ANKRD1*, *CTGF*, and *CYR61* genes in Con-, ST6GAL1-KO-, and ST6GAL1-Res- MDA-MB-231 cells were detected by quantitative real-time PCR (qPCR). Error bars in (*F*), and (*G*) are mean ± SD (*n* = 3 biological replicates, ∗∗, *p* < 0.01; ∗∗∗, *p* < 0.001, and ∗∗∗∗, *p* < 0.0001 are determined by one-way ANOVA with Tukey's *post hoc* test). LATS, large tumor suppressor kinase; YAP, yes-associated protein; ST6GAL1, β-galactoside α2,6-sialyltransferase 1.

As described above, ST6GAL1-mediated α2,6-sialylation can negatively regulate Hippo signaling. To further validate this phenotype in breast cancer cells, we initially established two different ST6GAL1-KO MDA-MB-231 pooled cell lines (KO1 and KO2) using CRISPR/Cas9-based vectors, which were constructed with two independent single guide RNAs (sgRNAs). We then examined the phosphorylation status of the LATS1 and YAP proteins. As shown in Figure 3*A*, the phosphorylation levels of LATS1 (T1079) and YAP (S127) were significantly upregulated after the deletion of ST6GAL1. We further choose KO1 as KO for the following studies. Significantly, the overexpression of ST6GAL1 in the KO cells (KO-ST6GAL1-rescue [Res]) greatly rescued the Hippo signaling similar to the Con MDA-MB-231 cells (Fig. 3, *B* and *D*). Almost the same phenotype was observed in the BT549 cells (Fig. 3, *C* and *D*). Together, these results indicate that the inhibition of ST6GAL1 leads to YAP inactivation in breast cancer cells.

To further confirm the effect of ST6GAL1 on YAP inactivation, we checked the YAP's localization. As shown in Figure 3*E*, YAP protein is predominantly localized in the nucleus of Con MDA-MB-231 cells, interestingly, deletion of ST6GAL1 resulted in the redistribution of YAP from nucleus to cytoplasm (Fig. 3*E*). Moreover, compared with Con MDA-MB-231 cells, ST6GAL1-KO cells exhibited lower activity of a YAP-TEAD luciferase reporter and lower mRNA levels of previously reported classical YAP target genes, including *ANKRD1*, *CTGF*, and *CYR61*, which can be primarily rescued in ST6GAL1-Res cells (Fig. 3, *F* and *G*). Importantly, the same phenotype was validated in the BT549 cells (Fig. S1, *A*–*C*). Collectively, these results demonstrate that the lack of ST6GAL1 results in the enhanced phosphorylation of YAP(S127) (which leads to its cytoplasmic sequestration and inactivation), indicating the critical role of ST6GAL1 in suppressing the Hippo pathway in breast cancer cells.

### ST6GAL1 expression is required for ST3GAL4-mediated activation of Hippo pathway

**Figure 4:**
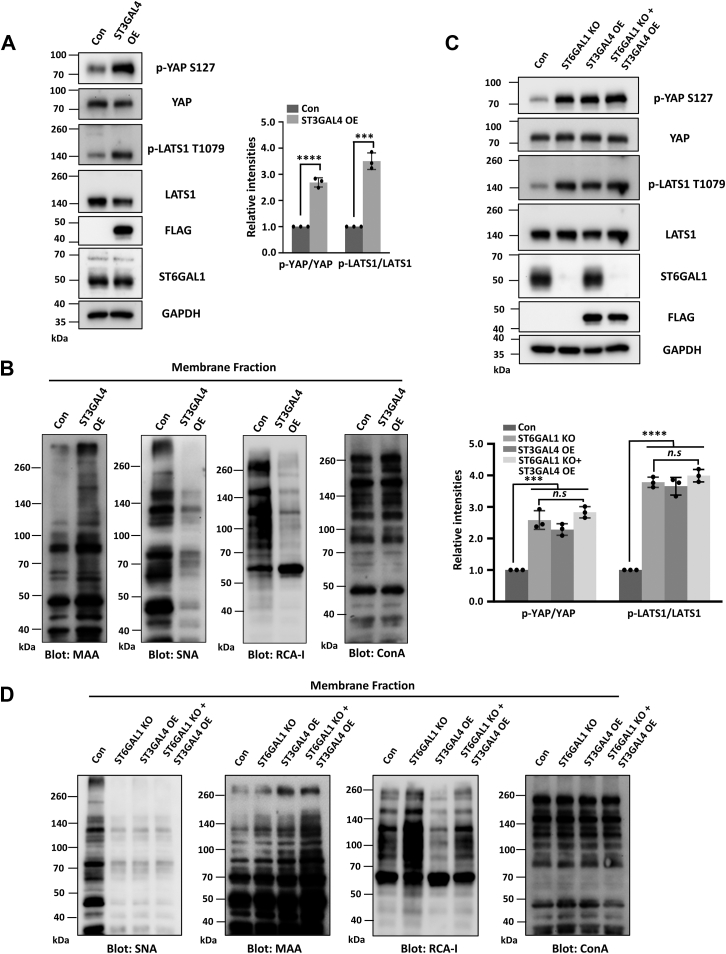
**α2,3-sialylation regulates the Hippo pathway through competing with α2,6-sialylation.***A,* the cell lysates from ST3GAL4-3xFLAG-overexpressing (ST3GAL4-OE) MDA-MB-231 cells were immunoblotted with anti-p-YAP S127, anti-YAP, anti-p-LATS1 T1079, anti-LATS1, anti-FLAG, anti-ST6GAL1, and anti-GAPDH antibodies. *B,* the cell membrane fractions from Con and ST3GAL4-OE cells were blotted with ConA, RCA-I (recognizing terminal galactose), MAA, and SNA lectins. *C,* cell lysates from the MDA-MB-231 derivative cell lines as indicated (The ST6GAL1 KO + ST3GAL4 OE stable cell line was established by overexpressing ST3GAL4 in ST6GAL1 KO cells.) were immunoblotted with indicated antibodies as mentioned in (*A*). The relative ratios (phospho-YAP and phospho-LATS1 *versus* YAP and LATS1) in (*A*) and (*C*) are shown as the mean ± SD (*n* = 3 biological replicates, *n.s.* not statistically significant, *p* > 0.05, ∗∗∗, *p* < 0.001, ∗∗∗∗, *p* < 0.0001 are determined by two-tail unpaired *t* test and one-way ANOVA with Tukey's *post hoc* test, respectively). *D,* the cell membrane fractions from the cells mentioned in (*C*) were blotted with ConA, RCA-I, MAA, and SNA lectins. LATS, large tumor suppressor kinase; SNA, *Sambucus nigra*; YAP, yes-associated protein; RCA-I, Ricinus communis agglutinin I; ConA, Concanavalin A; ST6GAL1, β-galactoside α2,6-sialyltransferase 1; MAA, *Maackia amurensis* agglutinin; Con, control.

Considering previous results showed that overexpression of ST3GAL4, which mainly catalyzes the α2,3-sialylation on *N*-glycans, promoted the activation of the Hippo pathway, we were wondering whether this was the case in breast cancer cells. Thus, we overexpressed ST3GAL4 in MDA-MB-231 cells, which was confirmed by Western blot (WB) and MAA- or Ricinus communis agglutinin I (RCA-I) lectin blot (Fig. 4, *A* and *B*). Consistently, ST3GAL4-OE cells exhibited a higher level of phosphorylated YAP(S127) and LATS1(T1079), as compared with the Con cells (Fig. 4*A*). Together, these results show that both the decreased expression of ST6GAL1 and the increased expression of ST3GAL4 exerted similar effects on the phosphorylation of LATS1(T1079) and YAP(S127), further demonstrating the key role of ST6GAL1 in suppressing the Hippo pathway.

Notably, we found that the reactivity of SNA-lectin was dramatically decreased after ST3GAL4 overexpression (Fig. 4*B*); however, ST3GAL4 did not affect the expression of ST6GAL1 (Fig. 4*A*). In addition, the reactivity of MAA lectin was dramatically increased after deletion of ST6GAL1 (Fig. 4*D*). Given that α2,6-sialyltransferases and α2,3-sialyltransferases share common substrates, we wondered that whether α2,6-sialylation and α2,3-sialylation could exhibit complementary functions on YAP signaling. To test it, the *ST3GAL4* gene was OE in the ST6GAL1-KO cells, which was confirmed by WB and MAA-, SNA-, or RCA-I lectin blot (Fig. 4, *C* and *D*). The results showed the forced expression of ST3GAL4 in ST6GAL1-KO cells did not result in further activation of the Hippo pathway, which indicated α2,6-sialylation, but not α2,3-sialylation, played significant roles in the inactivation of LATS1 and YAP (Fig. 4*C*). To further test this, we generated the ST3GAL4 knockdown cells in ST6GAL1-KO MDA-MB-231 cells, which was confirmed by WB and MAA- or RCA-I lectin blot (Fig. S2, *A* and *B*). As the result shown in Fig. S2*A*, knockdown of ST3GAL4 did not affect YAP phosphorylation, which further indicated that ST3GAL4 promoted Hippo pathway in a ST6GAL1 expression–dependent manner. Taken together, the above results indicate that the expression of ST6GAL1 is essential for the enhanced phosphorylation of LATS1(T1079) and YAP(S127) in ST3GAL4-OE cells, indicating the critical role of ST6GAL1 in promoting ST3GAL4-mediated α2,3-sialylation on Hippo pathway (which is majorly due to the decrease in ST6GAL1-mediated α2,6-sialylation *via* substrate competition mechanisms).

### YAP does not affect the expression of ST6GAL1 and cell surface sialylation

To evaluate the effects of YAP on the cell surface sialylation, we treated MDA-MB-231 cells with verteporfin (VP), an inhibitor of YAP/TEAD complex, which was identified to disrupt the association between YAP and TEAD family members, induce YAP cytoplasmic retention, degradation, and thus blocking the transcription of downstream targets of YAP (20). Cells were pretreated with different concentrations of VP and subject to WB analysis. As shown in Fig. S3*A*, VP treatment induced a dose-dependent decrease in YAP level, and 0.8 μM VP could markedly downregulate YAP expression in MDA-MB-231 cells. Furthermore, the cell membrane fractions of untreated and 0.8 μM VP-treated cells were blotted with MAA, SNA, and Concanavalin A (ConA) lectins, respectively. The lectin blot results showed the inhibition of YAP had little effect on both α2,3-sialylation and α2,6-sialylation in MDA-MB-231 cells (Fig. S3*B*). To confirm this result further, we established three cell lines with different YAP levels and activities: Con cells transfected with Con vectors; YAP-WT group stably overexpress WT YAP; YAP-S127A group stably overexpress constitutively activated YAP because of the mutation of serine 127 to alanine. As expected, an increased YAP level was observed in both YAP OE cells, and increased phosphorylation of YAP(S127) was observed in YAP-WT cells but not in YAP-S127A cells (Fig. S3*C*). Consistently, the Con and YAP-WT or YAP-S127A overexpression cells showed comparable expression of ST6GAL1, reactivity with MAA and SNA lectins (Fig. S3*D*). These results demonstrate that activated YAP cannot induce the upregulation of ST6GAL1, α2,3-sialylation, and α2,6-sialylation.

### ST6GAL1 mediates cell proliferation, migration, adhesion, and spreading through YAP activation

**Figure 5:**
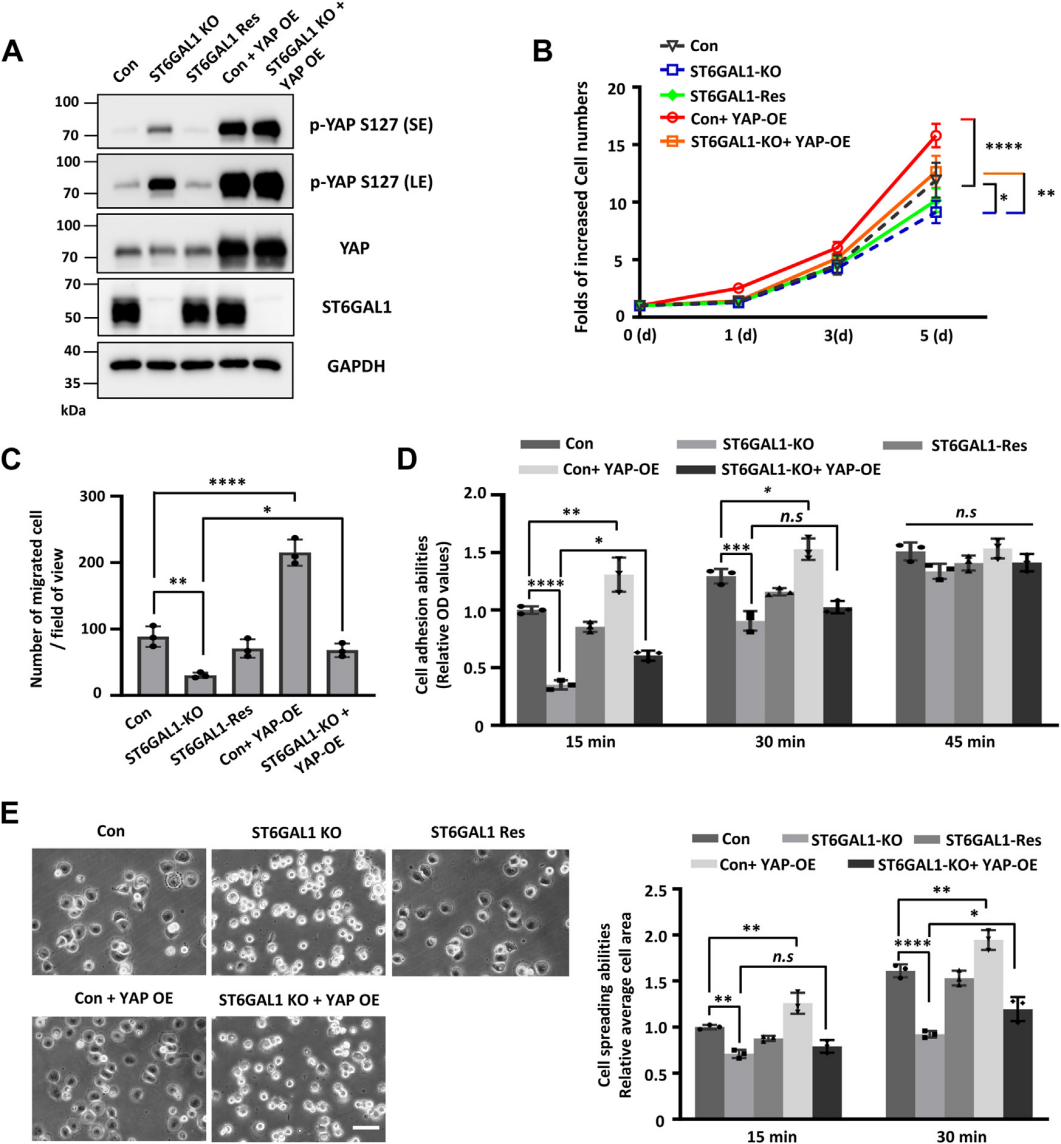
**The ST6GAL1–YAP axis is involved in growth, migration, adhesion, and spreading of MDA-MB-231 cells.***A,* the cell lysates from Con-, ST6GAL1-KO-, ST6GAL1-Res-, Con + YAP-OE-, and ST6GAL1-KO + YAP-OE- MDA-MB-231 cells were immunoblotted with anti-p-YAP S127, anti-YAP, anti-ST6GAL1, and β-actin antibodies. *B,* growth curve of indicated MDA-MB-231 cells mentioned in (*A*). *C,* FN-mediated transwell migration assays of indicated MDA-MB-231 cells mentioned in (*A*). *D,* indicated MDA-MB-231 cells adhesion abilities toward FN at different time-points (15, 30, and 45 min). *E,* cell spreading assay of indicated MDA-MB-231 cells mentioned in (*A*) spread on FN for 15 or 30 min. The scale bar represents 120 μm. The error bars in (*B–E*) are presented as the mean ± SD (*n* = 3 biological replicates, *n.s.* not statistically significant, *p* > 0.05; ∗, *p* < 0.05; ∗∗, *p* < 0.01; ∗∗∗, *p* < 0.001; and ∗∗∗∗, *p* < 0.0001 are determined by two-way ANOVA with Tukey's *post hoc* test in *panelB* or one-way ANOVA with Tukey's *post hoc* test in *panelC, D,* and *E*). LATS, large tumor suppressor kinase; YAP, yes-associated protein; ST6GAL1, β-galactoside α2,6-sialyltransferase 1; LE, long exposure; SE, short exposure; Con, control; Res, rescue; FN, fibronectin.

Next, to check the effects of ST6GAL1–YAP axis on cell biological functions in breast cancer cells, we OE YAP in both MDA-MB-231- and BT549-Con and ST6GAL1-KO cells (Fig. 5*A* and Fig. S3*A*). By cell growth assay and Boyden chamber analysis, we found that knockout of ST6GAL1 drastically inhibited both cell proliferation (Figs. 5*B* and S4*B*) and migration on fibronectin (FN) (Figs. 5*C* and S4*C*), which is consistent with the effects of ST6GAL1 in previous reports (21, 22). Significantly, re-expression of ST6GALT1 rescued the inhibitory effect of ST6GALT1 deficiency on cell proliferation and migration (Figs. 5, *B* and *C* and S4, *B* and *C*). Notably, although the phosphorylation expression level of YAP did not show much difference in ST6GAL1-KO + YAP-OE cell compared with the Con + YAP-OE cells (Figs. 5*A* and S4*A*), the promotional effect of YAP overexpression on cell proliferation and migration was largely canceled in ST6GAL1-KO + YAP-OE cell group (Figs. 5, *B* and *C* and S4, *B* and *C*), indicating ST6GAL1 is essential for YAP-mediated cell proliferation and migration and YAP overexpression can mask the effect of ST6GAL1 on Hippo signaling, which warrants future investigation to explore the interplay between ST6GAL1 activity and YAP–TEAD pathway.

To explore the underlying mechanisms involved in the ST6GALT1–YAP axis–mediated cell migration, we compared the cell adhesion and spreading abilities of Con-, ST6GAL1-KO-, ST6GAL1-Res-, Con + YAP-OE-, and ST6GAL1-KO + YAP-OE- MDA-MB-231 cells on FN. As shown in Figure 5, *D* and *E*, the cell adhesion (15 min time point) and spreading (30 min time point) ability of ST6GAL1-KO cells was significantly decreased when compared to Con cells, which can be primarily rescued in ST6GAL1-Res cells. Notably, the increased cell spreading ability after YAP overexpression was inhibited considerably in ST6GAL1-KO + YAP-OE cells (Fig. 5, *D* and *E*). In addition, the same cell spreading phenotype was observed in indicated BT549 cells (Fig. S4*D*). Taken together, ST6GAL1 is an adhesion-, spreading-, growth-, and migration-promoting GT, and its function is mediated, at least in part, by YAP activation.

### ST6GAL1 is essential for the activation of multiple upstream cell membrane receptors of the Hippo pathway

**Figure 6:**
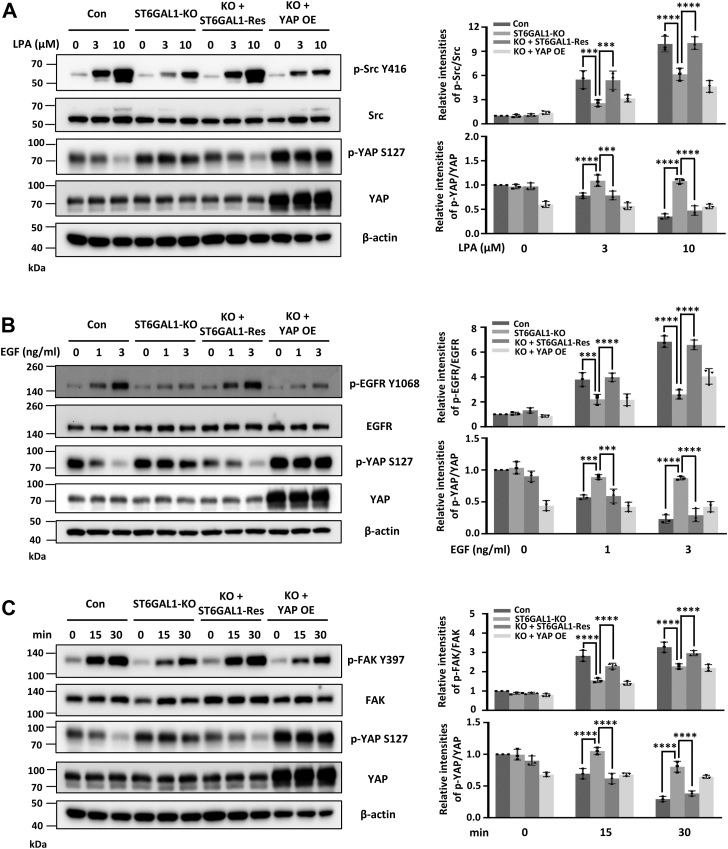
**ST6GAL1 is involved in the LPA, EGF, and FN-mediated YAP activation responses in MDA-MB-231 cells.***A* and *B,* after starvation for 36 h, the Con-, ST6GAL1-KO-, KO +ST6GAL1-Res-, and KO + YAP-OE- MDA-MB-231 cells were treated with LPA for 30 min (*A*) or EGF for 20 min (*B*) at the indicated concentrations. WB analysis was performed with anti-p-Src Y416, anti-Src, anti-p-YAP S127, anti-YAP, and anti-β-actin antibodies in *panel A* and anti-p-EGFR Y1068, anti-EGFR, anti-p-YAP S127, anti-YAP, and anti-β-actin antibodies in *panel B*. *C,* the indicated MDA-MB-231 cells were detached, suspended in assay medium for 40 min, and then replated onto an FN-coated plate for the indicated times. WB analysis was performed with anti-p-FAK Y397, anti-FAK, anti-p-YAP S127, anti-YAP, and anti-β-actin antibodies. The relative ratios (phospho-Src and phospho-YAP *versus* Src and YAP) in (*A*), (phospho-EGFR and phospho-YAP *versus* EGFR and YAP) in (*B*), and (phospho-FAK and phospho-YAP *versus* FAK and YAP) in (*C*) are shown as the mean ± SD (*n* = 3 biological replicates, ∗∗∗, *p* < 0.001; ∗∗∗∗, *p* < 0.0001 are determined by one-way ANOVA with Tukey's *post hoc* test). LATS, large tumor suppressor kinase; YAP, yes-associated protein; ST6GAL1, β-galactoside α2,6-sialyltransferase 1; EGF, epidermal growth factor; FAK, focal adhesion kinase; FN, fibronectin; EGFR, epidermal growth factor receptor; LPA, lysophosphatidic acid; EGF, epidermal growth factor; WB, Western blot; OE, overexpressed; Con, control.

Given that the Hippo pathway has been reported to be regulated by various cell membrane receptors and most cell membrane proteins are glycosylated (8, 15, 17, 23, 24), it is possible to speculate that ST6GAL1 is required for receptor glycoprotein-mediated inactivation of Hippo pathway. To test this, we checked the effect of ST6GAL1 on several membrane proteins-mediated YAP activation, including GPCRs, RTKs), and integrins (Fig. 6, *A*–*C*).

First, we treated the MDA-MB-231 cells with lysophosphatidic acid (LPA), an important extracellular signaling molecule that can trigger the YAP activation through GPCRs. As shown in Figure 6*A*, LPA treatment resulted in activation of Src signaling and suppression of the Hippo pathway and deletion of ST6GAL1, even further with YAP overexpression, exhibited delayed Src response and attenuated YAP dephosphorylation compared with those in Con cells. Restoration of ST6GAL1 enhanced both the Src and YAP response, which was similar to that in Con cells (Fig. 6*A*). In addition, epidermal growth factor (EGF) treatment resulted in the activation of EGFR signaling and enhancement of YAP phosphorylation; however, deletion of ST6GAL1 exhibited attenuated EGFR response and suppression of dephosphorylation of YAP (Fig. 6*B*). It is well known that integrins facilitate cell adhesion, spreading, and migration, as well as Hippo pathways, during which the activation of focal adhesion kinase (FAK) is a critical step (25). To determine whether ST6GAL1 affected the FN-mediated activation of FAK, serum-starved cells were replated onto FN-coated dishes and then harvested at the indicated times. The phosphorylation levels of FAK (p-FAK, Y397) in the cell lysates were detected. As expected, the levels of p-FAK upon FN stimulation at each time point in ST6GAL1-KO cells were lower than those in Con cells, and ST6GAL1-KO cells exhibited attenuated YAP dephosphorylation (Fig. 6*C*). These phenotypes can be rescued in ST6GAL1-Res cells (Fig. 6*C*). Importantly, consistent with the data of MDA-MB-231 cells, almost the same tendencies were observed in indicated BT549 cells (Fig. S5, *A*–*C*). Together, these results indicate that ST6GAL1 may suppress phosphorylation of YAP(S127) *via* GPCRs, RTKs, and integrins, which link the Hippo pathway to extracellular factors-receptors response system.

**Figure 7:**
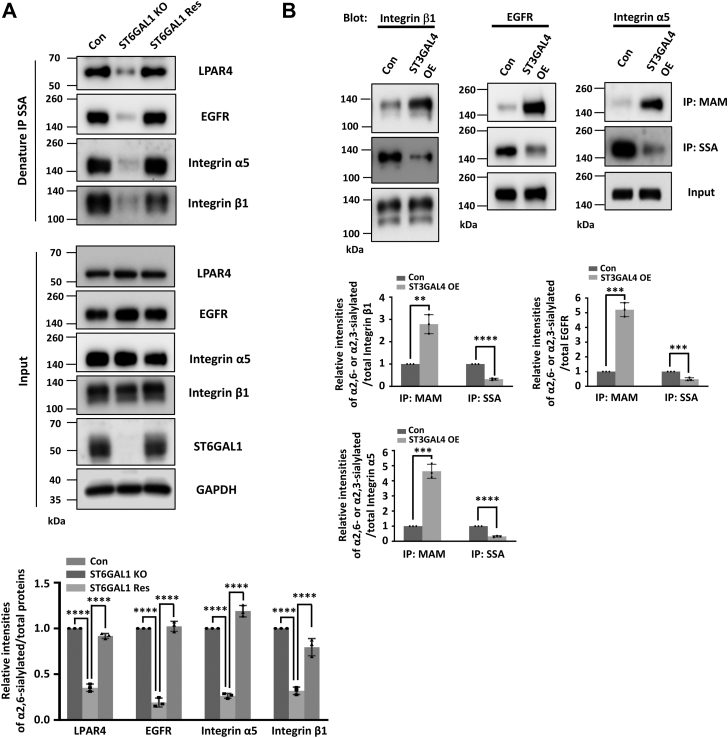
**ST6GAL1 catalyzes the α2,6-sialylation of LPAR4, EGFR, integrin α5, and integrin β1 in MDA-MB-231 cells.***A,* the cell lysates from Con-, ST6GAL1-KO-, ST6GAL1-Res- MDA-MB-231 cells were immunoprecipitated by SSA-agaroses and then blotted with antibodies against LPAR4, EGFR, integrin β1, and integrin α5. The whole-cell lysates were also subjected to WB with indicated antibodies. *B,* the cell lysates from Con- and ST3GAL4-OE- MDA-MB-231 cells were immunoprecipitated by SSA- and MAM-agaroses and then blotted with antibodies against integrin β1, EGFR, and integrin α5 separately. The whole-cell lysates were also subjected to WB with indicated antibodies. The relative ratios (the α2,6-sialylated LPAR4, EGFR, integrin α5, or integrin β1 *versus* total LPAR4, EGFR, integrin α5, or integrin β1, respectively) in (*A*) and (the α2,3-sialylated or α2,6-sialylated integrin β1, EGFR, or integrin α5 *versus* total integrin β1, EGFR, or integrin α5, respectively) in (*B*) are shown as the mean ± SD (*n* = 3 biological replicates, ∗∗, *p* < 0.01; ∗∗∗, *p* < 0.001; and ∗∗∗∗, *p* < 0.0001 are determined by one-way ANOVA with Tukey's *post hoc* test and two-tail unpaired *t* test, respectively). WB, Western blot; ST6GAL1, β-galactoside α2,6-sialyltransferase 1; SSA, *Sambucus sieboldiana* agglutinin; Con, control; Res, rescue; EGFR, epidermal growth factor receptor; *Maackia amurensis.*

To examine whether ST6GAL1 regulates the α2,6-sialylation on specific RTK, GPCR, and integrins proteins, we checked the expression of α2,6-sialylated LPAR4, EGFR, integrin α5, and integrin β1 in Con-, ST6GAL1-KO-, and ST6GAL1-Res- MDA-MB-231 cells. The *Sambucus sieboldiana* agglutinin (SSA) lectin-based immunoprecipitation results showed that knockout of ST6GAL1 significantly suppressed α2,6-sialylation on these receptors, but not the total expression levels (Fig. 7*A*). Importantly, the decreased α2,6-sialylation could be primarily restored in ST6GAL1-Res cells (Fig. 7*B*). Furthermore, we checked the relationship between α2,3- and α2,6-sialylations on these receptors, as expected, forced expression of ST3GAL4 led to a dramatic reduction of α2,6-sialylation on integrin β1, EGFR, and integrin α5 (Fig. 7*B*). Taken together, our results demonstrate that ST6GAL1-mediated α2,6-sialylation on LPAR4, EGFR, and integrin α5β1 is responsible for their promotional effect on YAP activation.

### ST6GAL1 is essential for the integrin β1–EGFR/LPAR4 complex formation

**Figure 8:**
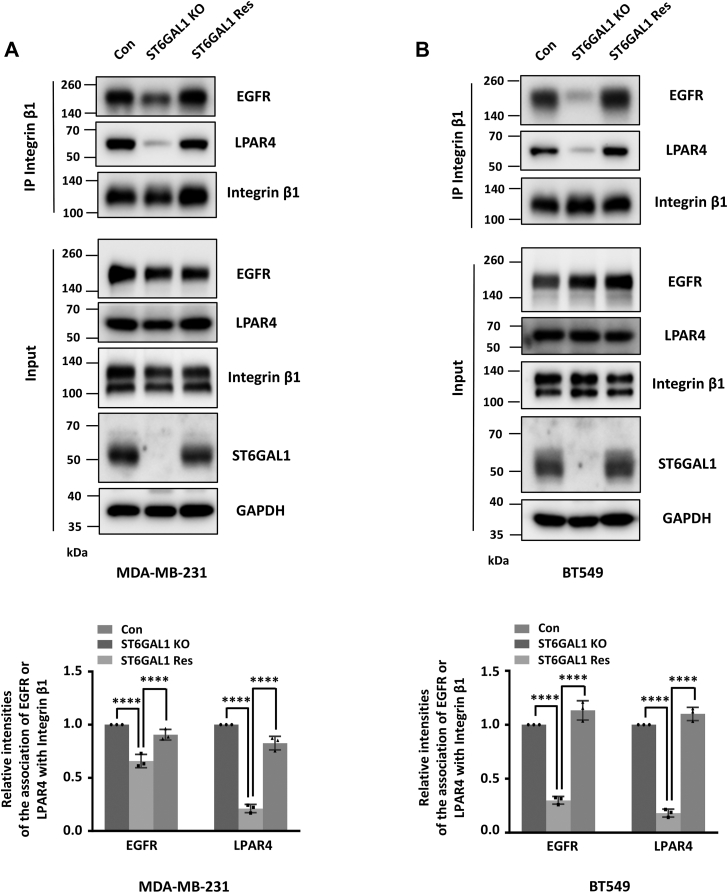
**ST6GAL1 mediates integrin β1–LPAR4/EGFR complex formation.***A* and *B,* the cell lysates from Con-, ST6GAL1-KO-, ST6GAL1-Res- MDA-MB-231 (*A*) and BT549 (*B*) cells were immunoprecipitated by anti-integrin β1 antibody and then blotted with antibodies against LPAR4, EGFR, and integrin β1. The whole-cell lysates were also subjected to WB with indicated antibodies. The relative ratios (the association of EGFR or LPAR4 with integrin β1, respectively) in (*A*) and (*B*) are presented as the mean ± SD (*n* = 3 biological replicates, ∗∗∗∗, *p* < 0.0001 is determined by one-way ANOVA with Tukey's *post hoc* test). ST6GAL1, β-galactoside α2,6-sialyltransferase 1; Con, control; Res, rescue; EGFR, epidermal growth factor receptor; WB, Western blot.

The above data led us to seek how ST6GAL1-mediated α2,6-sialylation participates in the response of multiple cell membrane receptors. Considering that integrins-mediated cellular signalings are thought to be regulated by cooperating with other membrane proteins, including RTKs and GPCRs (26, 27), we wondered whether integrin β1 is associated with LPAR4 and EGFR through α2,6-sialylation. Immunoprecipitates with anti-integrin β1 antibody showed that integrin β1 indeed interacted with LPAR4 and EGFR in MDA-MB-231 cells (Fig. 8*A*), which was consistent with previous reports (26, 28, 29). Interestingly, the interactions between integrin β1 and LPAR4/EGFR were significantly decreased upon ST6GAL1 deletion (Fig. 8*A*). Importantly, restoration of ST6GAL1 in ST6GAL1-KO MDA-MB-231 cells largely rescued the integrin β1–LPAR4/EGFR complex formation. Moreover, the decreased and rescued interactions between integrin β1 and LPAR4/EGFR were also observed in ST6GAL1-KO and ST6GAL1-Res BT549 cells (Fig. 8*B*). Taken together, these results suggest that the ST6GAL1-mediated α2,6-sialylation is required for the interaction between integrin β1 and LPAR4/EGFR, which may promote their responses to LPA, EGF, and FN stimulations and cell biological functions.

### ST6GAL1–YAP axis promotes the metastatic ability of breast cancer cells

**Figure 9:**
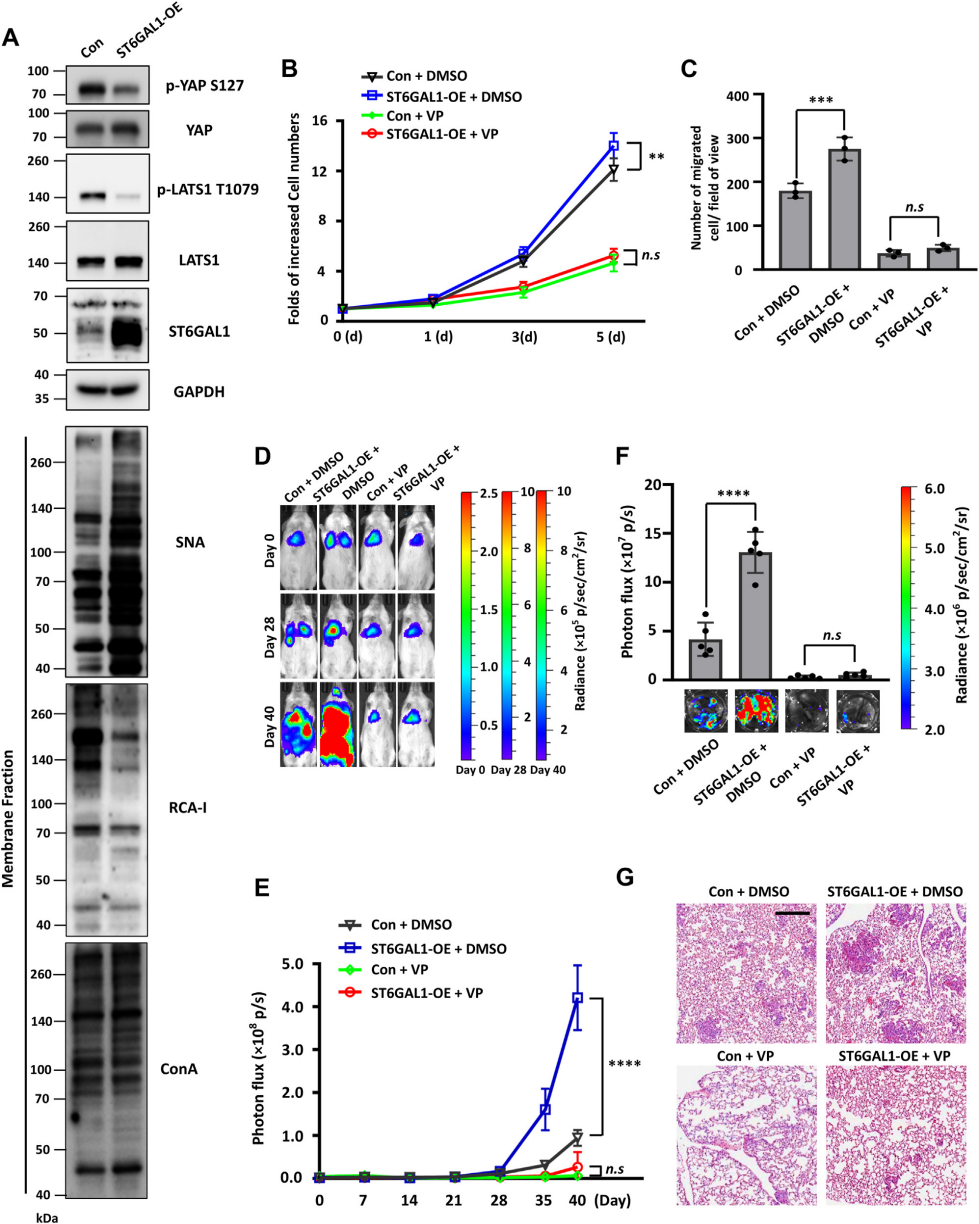
**ST6GAL1–YAP axis regulates the metastatic ability of breast cancer cells.***A,* the cell lysates from Con- and ST6GAL1-OE- MDA-MB-231-Luc cells were immunoblotted with anti-p-YAP S127, anti-YAP, anti-p-LATS1 T1079, anti-LATS1, anti-ST6GAL1, and anti- GAPDH antibodies. The cell membrane fractions from the indicated cells were blotted with ConA, RCA-I, and SNA lectins. *B* and *C,* comparison of cell growth (*B*) and migration (*C*) among Con-, ST6GAL1-OE-cells treated with DMSO or VP. The cell proliferation ability (*B*) and the migration ability toward FN (*C*) were detected. The error bars are presented as the mean ± SD (*n* = 3 biological replicates, *n.s.* not statistically significant, *p* > 0.05; ∗∗, *p* < 0.01, ∗∗∗, *p* < 0.001 are determined by two-way ANOVA with Tukey's *post hoc* test in *panelB* or one-way ANOVA with Tukey's *post hoc* test in *panelC*). *D* and *E,* bioluminescent imaging (*D*) and quantification of photon flux (*E*) of NSG mice with intravenous injection of Con- and ST6GAL1-OE- MDA-MB-231-Luc cells, followed by DMSO or VP treatment. Day 0: the day of tumor cell injection. *F* and *G,* bioluminescent imaging (F, *lower panel*), quantification of photon flux (F, *upper panel*), and H&E staining (*G*) of the lungs from mice described in (*D*). The scale bar represents 250 μm. The error bars in (*E*) and (*F*) are presented as the mean ± SD (*n* = 5 mice per group, *n.s.* not statistically significant, *p* > 0.05; ∗∗∗∗, *p* < 0.0001 are determined by two-way ANOVA with Tukey's *post hoc* test in *panel E* or one-way ANOVA with Tukey's *post hoc* test in *panelF*). LATS, large tumor suppressor kinase; YAP, yes-associated protein; ST6GAL1, β-galactoside α2,6-sialyltransferase 1; OE, overexpressed; ConA, Concanavalin A; RCA-I, Ricinus communis agglutinin I; VP, verteporfin; FN, fibronectin; SNA, *Sambucus nigra*; Con, control.

To further confirm the effects of the ST6GAL1–YAP axis on cell biological functions, we OE the ST6GAL1 in MDA-MB-231 cells, which was confirmed by WB and SNA-, or RCA-I lectin blot (Fig. 9*A*). As expected, the phosphorylation levels of LATS1(T1079) and YAP(S127) and the reactivity of RCA-I lectin were significantly decreased after ST6GAL1 overexpression (Fig. 9*A*). Then, we treated Con and ST6GAL1-OE MDA-MB-231 cells with VP, which disrupt the YAP/TEAD complex formation and therefore block the transcriptional activation of genes downstream of YAP. Cell growth assay and Boyden chamber analysis showed that ST6GAL1 significantly increased the cell proliferation and migratory abilities, and these abilities of Con and ST6GAL1-OE cells were dramatically suppressed, and the difference between Con and ST6GAL1-OE group was neutralized after VP treatment (Fig. 9, *B* and *C*). Importantly, the same phenotype of cell signaling, proliferation, and migration were observed in ST6GAL1-OE BT549 cells (Fig. S6, *A*–*D*). These data demonstrated that ST6GAL1 contributed to pro-proliferative and migratory cell behaviors by inhibiting YAP.

Finally, we checked the effects of the ST6GAL1–YAP axis on metastasis through an *in vivo* metastatic mouse model by tail vein injection. Bioluminescent imaging of live animals (Fig. 9, *D* and *E*) and whole lungs (Fig. 9*F*), as well as H&E staining of lung sections (Fig. 9*G*), revealed that overexpression of ST6GAL1 in MDA-MB-231 cells strongly promoted lung metastasis in mice, which was primarily inhibited by VP treatment (Fig. 9, *D*–*G*). These data provide *in vivo* proof that the ST6GAL1–YAP axis promotes the metastatic ability of breast cancer cells.

## Discussion

**Figure 10:**
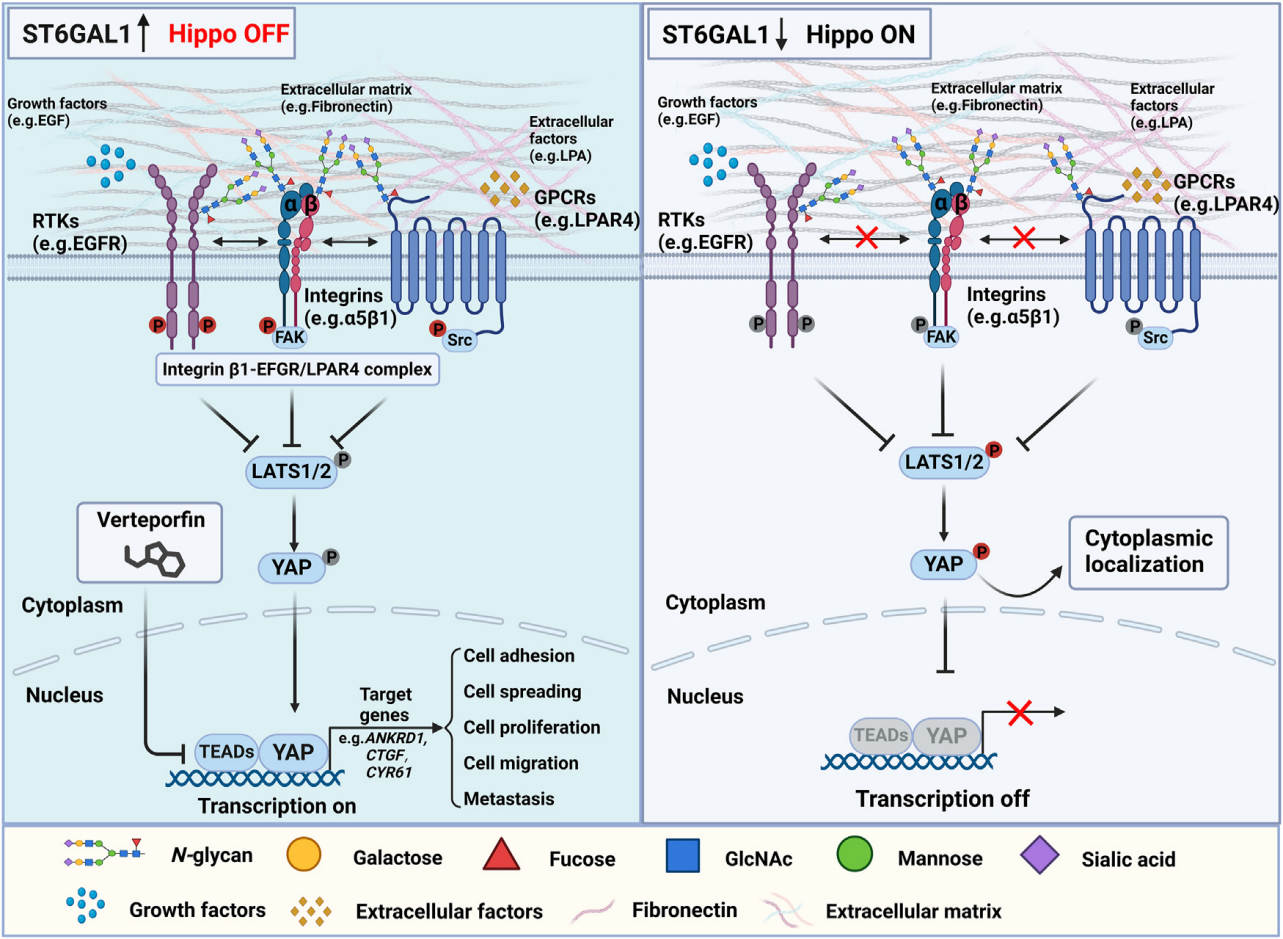
**Schematic diagram of the proposed molecular mechanism for negative regulation of Hippo signaling *via* ST6GAL1.** Various upstream cell membrane receptors of the Hippo pathway have been identified, including the RTKs (*e.g.*, EGFR), GPCRs (*e.g.*, LPAR4), and integrins (*e.g.*, integrin α5β1). The RTK, GPCR, and integrin signals transduced by growth factors (GFs, *e.g.*, EGF), extracellular factors (*e.g.*, LPA), and the extracellular matrix (ECM, *e.g.*, FN) can facilitate Hippo pathway effectors (*e.g.*, PI3K and FAK) association, which promote LATS1/2-mediated regulation of YAP. In the cells with ST6GAL1 expression (*left*), the cell membrane receptors, such as EGFR, LPAR4, and integrin α5β1, are modified by α2,6-sialylation, which mediate the integrin β1–EGFR/LPAR4 complex formation and in turn facilitate their responses to EGF, LPA, and FN, respectively. These signalings inactivate LATS1/2 kinases or induce the dephosphorylation of YAP, finally leading to hypophosphorylated YAP (p-YAP S127). Hypophosphorylated YAP accumulates in the nucleus, where it can bind to various transcription factors (TFs, *e.g.*, TEAD family) to enhance the expression of target genes (*e.g.*, *ANKRD1*, *CTGF*, and *CYR61*) expression that promote cell adhesion, spreading, proliferation, migration, and metastasis. The Hippo signaling can be inhibited by the verteporfin (VP) inhibitor, which targets YAP-TEAD activity. In the ST6GAL1 deficiency cells (*right*), the *N*-glycans on cell membrane receptors are without α2,6-sialylation, which exhibit weak integrin β1–EGFR/LPAR4 complex formation and delayed responses to EGF, LPA, and FN stimulation and activate the LATS1/2 kinases and phosphorylate YAP on S127. The phosphorylated YAP (p-YAP S127) is retained in the cytoplasm, inhibiting YAP/TEAD-dependent transcription. The p of the *red background* represents the activation of related proteins, while *gray background* represents the inactivation. LATS, large tumor suppressor kinase; YAP, yes-associated protein; ST6GAL1, β-galactoside α2,6-sialyltransferase 1; RTK, receptor tyrosine kinase; EGF, epidermal growth factor; EGFR, epidermal growth factor receptor; FN, fibronectin; GPCR, G protein–coupled receptor; GT, glycosyltransferase; LPA, lysophosphatidic acid; FAK, focal adhesion kinase.

The Hippo–YAP signaling pathway plays a pivotal role in organ size Con, and its deregulation promotes tumorigenesis. Although several upstream positive and negative pathway regulators have been identified in the past decade (30, 31), the potential molecular mechanisms remain unclear. In the present study, we outline the novel underlying mechanism responsible for the promoting effects of the ST6GAL1–YAP axis in breast cancer cell growth, migratory and adhesion ability *in vitro*, and metastasis *in vivo*. In detail, the ST6GAL1-catalyzed α2,6-sialylation of several Hippo upstream cell membrane receptors (including EGFR, LPAR4, and integrin α5β1) is responsible for integrin β1–EGFR/LPAR4 complex formation and their signal responses upon extracellular factor stimulation, resulting in the inactivation of Hippo pathway. Meanwhile, deletion of ST6GAL1 suppresses the complex formation and responses of these receptors, which leads to phosphorylation of the core Hippo effectors (LATS1 and YAP), resulting in the inactivation of the downstream oncoprotein (YAP). Furthermore, VP, an inhibitor of YAP/TEAD interaction, can counteract the roles of ST6GAL1 in breast cancer cells (Fig. 10). Consistent with these observations, Wang *et al.* reported that YAP/TEAD was essential to sustain the malignant behaviors of invasive breast cancer cells (32), which further highlight the importance of identifying the upstream regulators of Hippo pathway. Our data reveal that ST6GAL1 could affect the Hippo–YAP signaling, which provides new insights into the linkage between aberrant *N*-glycosylation and inactivated Hippo pathway in breast tumors.

An increasing body of evidence suggests the fundamental roles of ST6GAL1 in tumor malignant progression; a better understanding of the potential molecular mechanisms of this sialyltransferase is critical. Our findings showed that deletion of ST6GAL1 activated the Hippo tumor suppressor pathway in both MDA-MB-231 and BT549 cells, indicating a vital role of ST6GAL1 in the inactivation of Hippo signaling in breast cancer. In addition, we demonstrated that YAP did not affect the expressions of ST6GAL1 and α2,6-sialylation. It is worth noting that the promotional effect of ST6GAL1 on YAP activation was masked by YAP overexpression, however, the increased cell proliferation and migration abilities in Con cells after YAP overexpression were largely inhibited in absence of ST6GAL1. These findings suggested that ST6GAL1 was also essential for YAP-mediated cell biological functions, highlighting the crosstalk between ST6GAL1 and Hippo signaling, which need further studies to address this regulation.

It has been reported that the Hippo pathway was implicated in contact inhibition of proliferation; however, contact inhibition of proliferation was determined by the interplay between cells and the microenvironment (6, 33, 34). Given the outmost location of the sialic acids and the relatively strong electronegative charge, it is reasonable to postulate that the effects of ST6GAL1-mediated α2,6-sialylation on Hippo signaling could be attributed to the altered interplay between cells and microenvironment. It is well known that elevated expression of sialylation, especially α2,6-sialylation catalyzed by ST6GAL1, has been reported in multiple types of cancers and correlated with epithelial–mesenchymal transition, cell-matrix adhesion, migration, and tumor immune microenvironment (35, 36, 37, 38, 39, 40). Mechanistically, previous studies reported that sialylation on integrin β1 can promote cell adhesion, migration, and the phosphorylation of FAK by facilitating its activation (41, 42). Our study found that the role of ST6GAL1 in breast cancer is influenced by integrin-mediated Hippo signaling. Likewise, a previous study has revealed that upregulation of ST6GAL1 enhanced the caveolin-1–induced adhesive ability of hepatocellular carcinoma cells to FN *via* FAK signaling (43). However, this is not always the case. Suzuki *et al.* demonstrated that inhibition of ST6GAL1 expression dramatically enhanced lymphoma cell adhesion to galectin-8 (44). Although it is difficult to thoroughly explain the functions of ST6GAL1 in cell–cell and cell–matrix interactions, two aspects cannot be ignored. First, different cancer cell lines, extracellular matrices (ECMs) or the heterogeneity in 2,6-linked sialic acids could contribute to the variations in adhesion capacity; in detail, the breast cancer cells with medium level of α2,6-sialylation showed greater adhesion to reconstituted ECMs than cells expressing higher levels of α2,6-sialylation (45) and ST6GAL-mediated α2,6-sialylation could enhance cell adhesion to FN, collagen l, collagen IV, and laminin while inhibiting cell adhesion to galectin-3 in different colon cancer cell lines (36, 46, 47, 48, 49). Second, since the outmost reaches of sialic acids on *N*-glycans and their electronegative charge, cell surface glycan structure dramatically impacts the interactions between cells and the extracellular matrix. Collectively, due to the complexity of *N*-glycosylation, underlying mechanisms by which ST6GAL1 affects adhesion require further study.

As we know, both ST6GAL1 and ST3GAL4 catalyze the transfer of sialic acid to the common *N*-glycan substrate. Interestingly, our results showed that the α2,3-sialylation level was increased after ST6GAL1 deletion, and the α2,6-sialylation level was decreased in ST3GAL4 overexpression cells, revealing the competition role of ST6GAL1 and ST3GAL4 to catalyze sialylation. In addition, it is worth mentioning that overexpression or knockdown of ST3GAL4 in ST6GAL1-KO cells did not affect YAP phosphorylation as compared with ST6GAL1-KO cells, however, ST3GAL4 overexpression can active the Hippo signaling in parental cells. These results indicate that the promotional effect of ST3GAL4 on Hippo signaling is ST6GAL1 expression-dependent, highlighting the importance of ST6GAL1-mediated α2,6-sialylation, but not ST3GAL4-mediated α2,3-sialylation, in regulating Hippo signaling. Our observations raise the question of the mechanisms of the discrepancy in their function in regulating the Hippo signaling. One possible explanation is the effects of ST6GAL1 on specific glycoproteins, which are also implicated in the Hippo signaling and cancer cell behaviors, such as integrin β1, EGFR, and E-cadherin (6, 33, 50). In contrast, little influence of ST3GAL4 in these fields has been reported. The same phenomenon was observed by Feng *et al.* in HeLa cells that the α2,6-sialylation levels of EGFR were increased upon ST3GAL6 knockdown (51). This is probably because the GlcNAc branching of *N*-glycans may influence salic acid attachment. Previous studies showed that a low degree of branching favors α2,6-sialylation but does not favor α2,3-sialylation (52). Despite the similar size and negative charge of the sialic acids, the enzymatic products of ST6GAL1 and ST3GAL4 form different glycoprotein tertiary structures with distinct biological functions by adding the sialic acids to the various locations of terminal *N*-glycans. Therefore, identifying the crystal structure of such enzymatic products may provide a plausible explanation for the detailed mechanism.

In addition to EGFR, LPAR4, and integrin α5β1, various other membrane proteins upstream of the Hippo pathway can be glycosylated. Previous studies have reported that ST6GAL1 can modify ErbB2 to regulate cell proliferation and drug resistance (53, 54). In addition, Shashidhar *et al.* demonstrated that GPR56, a newly discovered orphan GPCR of the secretin family, can be modified by glycosylation, which further regulates cell adhesion (55). Also, the formation of *N*-glycans catalyzed by GnT-Ⅲ, GnT-V, and FUT8 plays an important role in the adhesion function of E-cadherin (56). However, the effect of glycosylation modifications of these membrane proteins on the Hippo pathway remains unknown, which may become the next objective. Given that the *N*-glycosylation of many proteins has not been researched thoroughly, more efforts should be put into detecting membrane proteins, whose glycosylation exerts an essential role in tumor progression.

Given the evidence that integrins-associated signalings are considered to cooperate with other membrane receptors (57) and the findings from our group that the specific *N*-glycans on integrins, especially integrin α5, β1, and β4, are essential for their mediated complex formation with several membrane proteins (58, 59, 60, 61, 62), it is reasonable to think that α2,6-sialylation may affect the interactions between integrin β1 and EGFR or LPAR4, controlling their mediated Hippo signaling. Here, we found and validated that ST6GAL1-mediated α2,6-sialylation was essential for the integrin β1–LPAR4/EGFR complex formation in both MDA-MB-231 and BT549, however, the effect of the crosstalk between Integrin β1 and LPAR4 or EGFR on Hippo signaling need further investigation.

The next issue concerned the question of how α2,6-sialylation mediates the Integrin β1-LPAR4/EGFR association. Considering our group's findings that lipid rafts can serve as platforms for the complex formation of *N*-glycosylated membrane proteins (including integrin α5/integrin β4–EGFR complexes) (61, 62), it is worth to identify the role of α2,6-sialylation in regulating the integrin β1–LPAR4/EGFR complex localization in lipid raft. However, we could not exclude the direct interaction through the potential unknown lectin-like domain on integrin β1, LPAR4, or EGFR, since integrin αMβ2 can interact with GlcNAc through its lectin domain (63). Notably, our group previously identified that α2,6-sialylation on the membrane-proximal domain of the integrin β1 mutant could facilitate its association with syndecan-4 and EGFR (60), therefore, it is tempting to explore the individual α2,6-sialylated *N*-glycan(s) and the detailed structure(s) on integrin β1, LPAR4, or EGFR, which is involved in the integrin β1–LPAR4/EGFR complex formation to integrate both the ECM and cytokines in the tumor microenvironment.

In most studies, ST6GAL1 was generally thought to be associated with more metastatic progression of many types of cancer, while the opposite conclusion has recently been reported (64, 65). Specifically, α2,6-sialylation of adhesion markers (including PODXL, ICAM1, ECE1, ALCAM1, CD97, and CD44) can inhibit cluster formation of circulating tumor cells and lung metastasis in triple-negative breast cancer (64). Besides, an increase in α2,6-sialylation of melanoma cell adhesion molecule inhibits its interaction with galectin-3 and decreases its expression on the cell surface, exerting hepatocellular carcinoma metastasis (65). We speculate that the contradictory correlation of α2,6-modification with tumor metastasis may be related to the following reasons (1): ST6GAL1 plays distinctive roles in a cancer type– and tissue type–specific manner (2); Tumor metastasis involves multiple phases, including ECM remodeling, intravasation, adhesion, extravasation, dormancy, distant seeding, and lymph node metastasis, ST6GAL1 may exert different functions at certain steps (66); and (3) downregulation of ST6GAL1 or α2,6-sialylation may increase the level of other types of *N*-glycosylation which can promote the metastasis and progression of the tumor. Given the complexity, multistep, and deeply heterogeneous nature of tumor metastasis, the role of ST6GAL1 in metastasis deserves further investigation.

Taken together, our study showed that ST6GAL1 was an upstream negative regulator of the Hippo pathway in breast cancer cells *via* sialylated proteins. Although the detailed mechanisms remain further understanding, we believe that linking sialylation to the Hippo signaling provides new insight into glycosylation and could explain how the cell surface receptor glycoproteins are integrated to regulate tumor cell behaviors.

## Experimental procedures

### Antibodies and reagents

The experiments were performed using the following antibodies: Rabbit antibodies against p-YAP(S127) (#13008S), p-LATS1(T1079) (#8654S), LATS1 (#3477S), p-Src(Y416) (#2101S), p-FAK(Y397) (#8556S), FAK (#3285S), EGFR (#4267S), p-EGFR(Y1068) (#3777S), and integrin β1 (#9699S) were from Cell Signaling Technology; mouse mAb against GAPDH (#sc-365062), and β-actin (#sc-47778) were from Santa Cruz Biotechnology; mouse mAb against integrin α5 (610633) was from BD Biosciences; rabbit pAbs against LPAR4 (22165-1-AP) and mouse mAb against YAP (66900-1-Ig) were obtained from Proteintech; rabbit pAb against ST3GAL4 (NBP1-69565) was obtained from Novus Biologicals; mouse mAbs against FLAG (clone M2, #F3165) and Src (clone GD11, #05-184) were from Sigma; goat pAb against ST6GAL1 (AF5924) was from R&D Systems; mouse mAb against integrin β1 (P5D2) was from Developmental Studies Hybridoma Bank. The peroxidase-conjugate goat antibodies against mice and rabbits were obtained from Promega and Cell Signaling Technology. The peroxidase-conjugated rabbit antibody against goat was obtained from R&D Systems. Alexa Fluor 488 goat anti-mouse IgG and streptavidin conjugate Alexa Fluor 647 were obtained from Invitrogen (Thermo Fisher Scientific). Biotinylated SNA, MAA, and ConA lectins were purchased from Seikagaku Kogyo Inc.; (RCA-I was from Vector Laboratories Inc.; *M. amurensis* agarose and SSA agarose were from J-Oil Mill. The TO-PRO-3 was from Molecular Probes; EGF, LPA, FN, and (VP were obtained from Sigma; protein-A/G PLUS beads were purchased from Santa Cruz Biotechnology; the Sialidase (neuraminidase) was from Nacalai Tesque; the TRIzol and dimethyl sulfoxide (DMSO) was obtained from Thermo Fisher Scientific; the D-luciferin was from PerkinElmer. The protease inhibitors and phosphatase inhibitors were from Nacalai Tesque.

### Cell lines and cell culture

HEK293T cells were purchased from the Cell Bank of the Chinese Academy of Sciences. The MDA-MB-231 and BT549 cell lines were purchased from the American Type Culture Collection. All the cell lines were authenticated by short tandem repeat profiling and confirmed to be free from *mycoplasma*. HEK293T and MDA-MB-231 cells were maintained in Dulbecco's modified Eagle's medium (DMEM), and the BT549 cell line was maintained in RPMI 1640 medium, supplemented with 10% fetal bovine serum (FBS), except for stimulation, cell growth, adhesion, spreading, and migration assays.

### Vectors

The CRISPR/Cas9-based ST6GAL1-KO vectors were established as described previously (67). Briefly, the sgRNA-specifying oligonucleotide sequences spanning human ST6GAL1 (CATTCGCCTGATGAACTCTC on exon 2 and CAGATGGGTCCCATACAATT on exon 3) were chosen from the human KO library sgRNAs (68). After annealing, the double-stranded guide oligonucleotides were cloned into the pSpCas9(BB)-2A-GFP (Addgene plasmid ID: 48138) vector.

The pLKO.1-puro lentiviral vector was used to generate the ST3GAL4 knockdown vectors. Inserted oligonucleotide sequences were listed as follows: shRNA#1 against *ST3GAL4*, 5′-CCTGGTAGCTTTCAAGGCAAT-3′, and shRNA#2 against *ST3GAL4*, 5′-GCAATGGACTTCCACTGGATT-3′.

For overexpression vectors, the pCDH-ST6GAL1 (a vector with relatively low expression of ST6GAL1, for Res experiment), pLenti-CMV-ST6GAL1 (a vector with relatively high expression of ST6GAL1, for overexpression experiment), pLenti-CMV-YAP, pLenti-CMV-YAP-S127A, and pLenti-CMV-luciferase-RFP vectors were generated in our laboratory by using the In-Fusion kit (Takara Bio) and Gateway cloning system kit (Thermo Fisher Scientific). The 3 × FLAG-tagged GTs pLVX-Puro vectors (including MGAT1, MGAT2, MGAT3, MGAT4A, MGAT4B, MGAT4C, MGAT4D, MGAT5, B4GALT1, FUT8, ST3GAL3, ST3GAL4, ST3GAL6, ST6GAL1, ST6GAL2, ST8SIA2, and ST8SIA4) were generated by Synbio Technologies.

### Virus production, infection, and stable cell lines construction

Virus production and infection were performed as described previously (69). In brief, the lentivirus-based GTs, YAP, luciferase overexpression, or ST3GAL4 knockdown vectors were cotransfected with packaging plasmids (psPAX2 and pMD2.G) into HEK293T cells. After transfection for 48 h, the lentivirus supernatants were collected. The indicated cells were infected with the resultant viral supernatant for 48 h and then selected by hygromycin B, puromycin, blasticidin, or cell sorting to get indicated stable cells.

The CRISPR/Cas9-based ST6GAL1-KO cells were established as described previously (67). Two sgRNA independent pSpCas9(BB)-2A-GFP-sgST6GAL1 vectors were separately transfected into the indicated cell lines by electroporation according to the manufacturer's instructions. The GFP-positive cells were sorted by FACSAria II (BD Bioscience) after culture for 3 days. The SNA-negative cells were sorted three times during the following 2-week culture. The pooled ST6GAL1-KO cells were confirmed by Western blot analyses as described below. After virus infection, the luciferase-RFP stably OE MDA-MB-231 cells were sorted three times by FACSAria II. Stable cell lines were used in subsequent studies.

### Membrane protein isolation

The cell membrane protein fractions were isolated by Thermo Fisher Scientific Mem-PER Plus Membrane Protein Extraction Kit (catalog no: 89842) according to the manufacturer's instructions.

### WB and immunoprecipitation

For WB, when indicated cells grow to the medium density (cell confluency was at ∼60%), the cells were washed with ice-cold phosphate-buffered saline (PBS) and lysed in lysis buffer (20 mM Tris-HCl pH 7.4, 150 mM NaCl, 1% Triton X-100) with protease inhibitors and phosphatase inhibitors for 30 min. The supernatants were collected by centrifugation at 15,000 rpm for 10 min at 4 °C, and protein concentrations were determined using a bicinchoninic acid protein assay kit (Pierce). An equal amount of protein lysates was subjected to SDS-PAGE and then transferred to an NC membrane (Bio-Rad). The membrane was detected with indicated primary antibodies or biotinylated SNA, MAA, RCA-I, and ConA lectins. The protein bands were visualized using secondary antibodies or Vectastain ABC kit (Vector Laboratories) and Immobilon Western enhanced Chemiluminescent HRP Substrate (Thermo Fisher Scientific) according to the manufacturer's instructions.

For lectin immunoprecipitation (IP), cells were lysed with lysis buffer (20 mM Tris–HCl pH 7.4, 150 mM NaCl, 1% Triton X-100) with protease inhibitors, phosphatase inhibitors, and 1% SDS for 30 min, and then heated at 95  °C for 5 min for denaturing, followed by a 10-fold dilution with lysis buffer (to 0.1% SDS) and sonication. The supernatant was collected by centrifuging, and the protein concentrations were determined using a bicinchoninic acid protein assay kit. Equivalent amounts of the supernatants were immunoprecipitated by *M. amurensis* agarose or SSA agarose for 1 h at 4 °C with rotation. Then, the immunoprecipitates were washed twice with lysis buffer and subjected to SDS-PAGE.

For endogenous IP, the indicated MDA-MB-231 and BT549 cells were lysed in NETN buffer (20 mM Tris–HCl, pH 8.0, 100 mM NaCl, 1 mM EDTA, 0.5% Nonidet P-40) with protease and phosphatase inhibitors for 1 h at 4 °C. The lysates were centrifuged, and the supernatants were precleared with protein-A/G PLUS beads at 4 °C for 30 min with rotation, followed by incubation with an antibody against integrin β1 at 4 °C for 2 h, and were then incubated with protein-A/G PLUS beads at 4 °C overnight. The beads were washed with NETN buffer three times and the immunoprecipitates were detected by WB.

For the related quantifications of Western bands, the data were quantified using ImageJ software and derived from three independent experiments.

### Luciferase reporter assay

The luciferase reporter assay was performed as described previously (70). Briefly, the indicated MDA-MB-231 cells were co-transfected with an 8 × GTIIC luciferase reporter (Addgene plasmid ID: 34615) and a TK-Renilla luciferase reporter (Promega, #E2241). 48 h after transfection, the reporter activities were measured using a Dual-Luciferase Reporter Assay system (Promega, #E1910) on a Gen5 Microplate Reader (BioTek).

### RNA isolation and qPCR

Total RNA was isolated using TRIzol reagent and then reverse transcribed with an iScript complementary DNA Synthesis Kit (Bio-Rad). The resulting complementary DNA was used for real-time PCR using the iTaq Universal SYBR Green Kit (Bio-Rad). β-Actin was used as an internal Con. Real-time PCR and data collection were performed on a CFX96 instrument (Bio-Rad). The primer sequences are provided as follows: *ANKRD1*-Forward 5′-CACTTCTAGCCCACCCTGTGA-3′; *ANKRD1*-Reverse 5′-CCACAGGTTCCGTAATGATTT-3′; *CTGF*-Forward 5′-CCAATGACAACGCCTCCTG-3′; *CTGF*-Reverse 5′-GAGCTTTCTGGCTGCACCA-3′; *CYR61*-Forward 5′-AGCCTCGCATCCTATACAACC-3′; *CYR61*-Reverse 5′-GAGTGCCGCCTTGTGAAAGAA-3′; *ACTB*-Forward 5′-GATCATTGCTCCTCCTGAGC-3′; *ACTB*-Reverse 5′-ACTCCTGCTTGCTGATCCAC-3′.

### Flow cytometric analysis

Indicated cells were grown to about 90% confluence and detached from the culture dishes using trypsin containing 1 mM EDTA, washed with ice-cold PBS, and stained with the biotinylated SNA lectin, which preferentially recognizes the α2,6-sialylated products or MAA which preferentially recognized α2,3-sialylated products, for 40 min on ice, followed by incubation with streptavidin-conjugated Alexa Fluor 647 (Invitrogen) for 30 min on ice. Finally, the cells were washed three times with PBS and analyzed *via* FACSCalibur flow cytometry (BD Biosciences).

### Cell growth assay

Indicated cells were seeded into 60-mm dishes overnight and then serum-starved for 24 h. After starvation, the cells were released with 5% FBS media with or without 0.4 μM VP, an inhibitor of the YAP/TEAD complex, for indicated time. The cells of the indicated time points were digested and stained with trypan blue, and the numbers of living cells were counted. Cell numbers were normalized to those at 0 h and statistically analyzed.

### Cell migration assay

Cell migration was examined by Transwell (BD BioCoat TM Con inserts, 8.0-mm inserts; BD Biosciences). Briefly, the transwell inserts were coated on the bottom side with 10 μg/ml FN at 4 °C overnight. Cells were pretreated with or without 0.4 μM VP for 24 h, trypsinized, and suspended in DMEM containing 10% FBS. The suspended cells were washed twice with PBS, and the supernatants were removed using a centrifuge. Cells were resuspended with serum-free DMEM or RPMI 1640 medium and diluted to 1 × 10^5^ cells/ml. To each FN-coated transwell insert, 500 μl aliquots of the cell suspension were added; then, the cells were incubated at 37 °C for 4.5 h. After incubation, cells on the upper side were removed by scraping with a cotton swab. The membranes of each transwell insert were fixed with 4% paraformaldehyde and stained with 0.5% crystal violet overnight. Cells migrated to the lower side were counted using a phase-contrast microscope.

### LPA and EGF stimulation response assays

The indicated MDA-MB-231 cells (3 × 10^4^) were seeded in 6-cm dishes overnight and then serum-starved for 36 h to assay the cell signal response upon LPA and EGF stimulation. After starvation, the cells were supplied with DMEM containing indicated concentrations of LPA (0, 3, and 10 μM) for 30 min or EGF (0, 1, 3 ng/ml) for 20 min, and then the cells were collected, lysed, and then analyzed by WB, as described above.

### Cell adhesion, spreading, and adhesion response assays

The 48-well plates, 6-well plates, or 6-cm dishes were coated with FN (10 μg/ml) in PBS at 4 °C overnight and then blocked with 1% bovine serum albumin (BSA) in DMEM for 1 h at 37 °C. The indicated MDA-MB-231 or BT549 cells were detached and suspended in assay medium (50 mM Hepes and 0.1% BSA in serum-free DMEM [pH 7.4]) for 45 min at 3 × 10^4^ cells/ml. For cell adhesion assay, the cell suspensions were allowed to attach to coated 48-well plates for 15, 30, or 45 min incubation. The nonadherent cells were gently removed by washing with PBS, adherent cells were stained, extracted, and then measured at 560 nm wavelength in a plate reader. For cell spreading assay, the cells were attached to coated 6-well plates for 15 or 30 min incubation, nonadherent cells were gently removed, attached cells were fixed with 4% paraformaldehyde for 15 min. The photos were taken, and the overall areas of the adhesion cells were measured by ImageJ software (https://imagej.net/ij/). For cell adhesion response assay, the suspensions were replated onto coated 6-cm dishes for 0, 15, and 30 min; the attached cells were collected, lysed, and then analyzed by WB.

### Immunofluorescence staining

Indicated cells cultured on glass-bottom dishes were fixed with methanol for 30 min at room temperature and then permeabilized with 0.2% Triton-X-100. A nonspecific blocking solution was applied (PBS, 0.1% Triton X-100, 10% BSA) at room temperature for 1 h, followed by incubation with antibody against YAP, then incubated with anti-mouse Alexa Fluor 488 secondary antibodies (Invitrogen) and TO-PRO-3 in the dark. Fluorescence images were observed *via* confocal microscopy using a FluoView FV1000 (Olympus). To better distinguish the color and the present staining results, we have replaced the overall color of YAP (green to red) and nuclei (red to blue) in the confocal software.

### Experimental metastasis assays

5 × 10^5^ indicated MDA-MB-231-Luc cells were injected into the tail vein of 6- to 8-week-old female NSG mice. After injection, we confirmed tumor cell engraftment by luciferase imaging of live animals using an IVIS-200 bioluminescence imaging system (PerkinElmer) after intraperitoneal injection of 100 μl D-luciferin substrate (25 mg/ml in PBS, PerkinElmer), excluded the outliers, and randomly divided the Con and ST6GAL1-OE mice into four treatment groups: (1) Con + DMSO (2); ST6GAL1-OE + DMSO (3); Con + VP; and (4) ST6GAL1-OE + VP. The treatment of VP was administered through the tail vein twice weekly at a dose of 1 mg/kg body weight till the endpoint. Metastasis was monitored by bioluminescent imaging. Mice were euthanized when they met the institutional euthanasia criteria for an overall health condition. The lungs were collected, imaged with D-luciferin substrate (150 μg/ml in PBS), and then processed for histopathological analysis. All animal procedures were carried out according to experimental protocols approved by the Tohoku Medical and Pharmaceutical University and Yangzhou University Research Ethics Board.

### Statistical analysis

Statistical analyses were performed using a one-way ANOVA with Dunnet's or Tukey's *post hoc* test, two-tail unpaired *t* test, or two-way ANOVA with Tukey's *post hoc* test depending on the indicated experiment situation *via* GraphPad Prism 10 (https://www.graphpad.com/). Results are presented as the mean ± SD. Statistical significance was defined as *n.s.* (not statistically significant), *p* > 0.05; ∗, *p* < 0.05; ∗∗, *p* < 0.01; ∗∗∗, *p* < 0.001; and ∗∗∗∗, *p* < 0.0001.

## Data availability

All data needed to evaluate the conclusions are present in the article.

## Supporting information

This article contains supporting information.

## Conflict of interest

The authors declare that they have no conflicts of interest with the contents of this article.
