# Supplementary material for: Inhibitory effects of β-galactoside α2,6-sialyltransferase 1 on the Hippo pathway in breast cancer cells
Source: J Biol Chem. 2025 May 21;301(10):110266. doi: 10.1016/j.jbc.2025.110266 (PMC12550800; doi:10.1016/j.jbc.2025.110266)

Supplementary Fig. 1

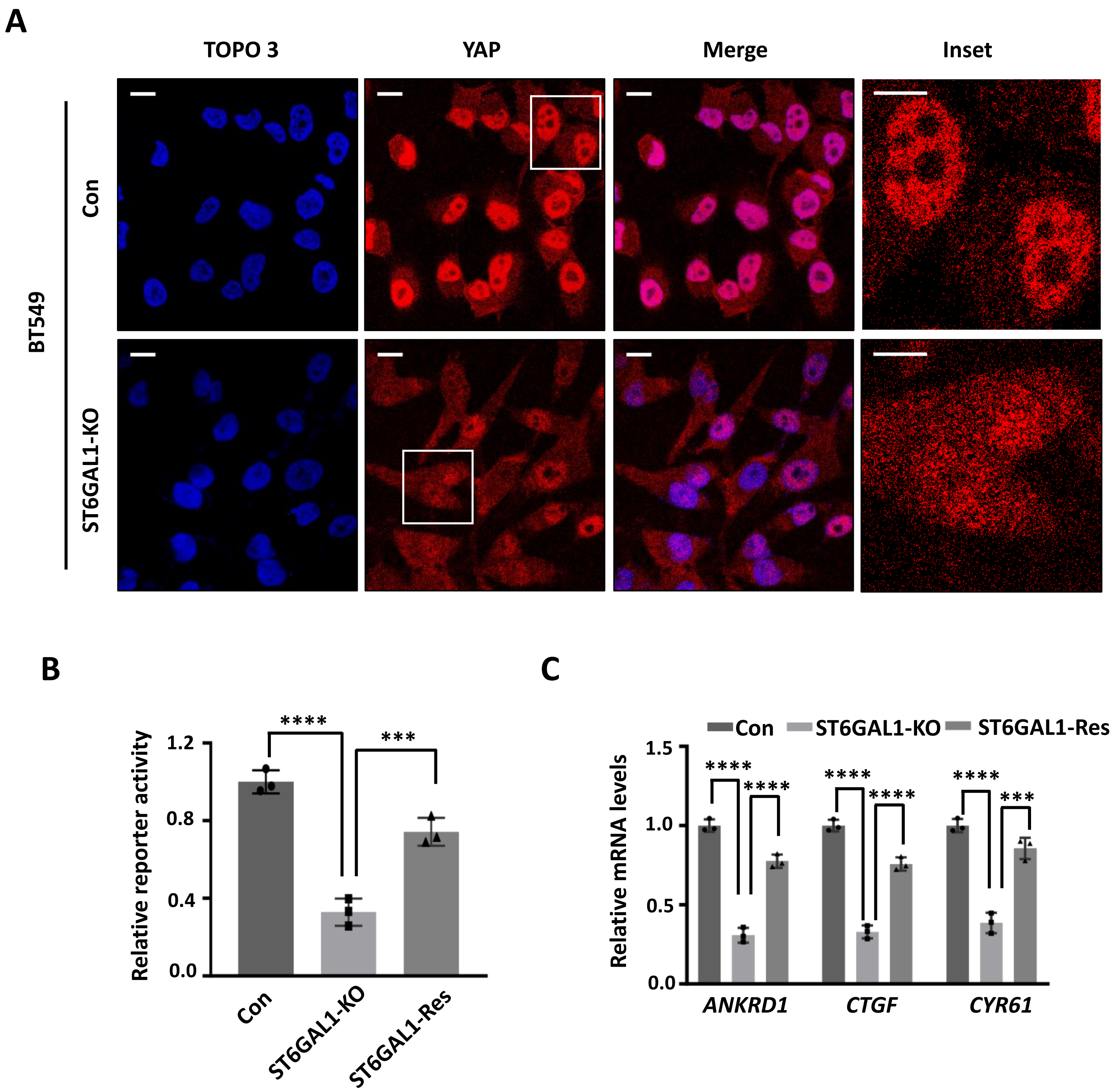

Supplementary Fig. 2

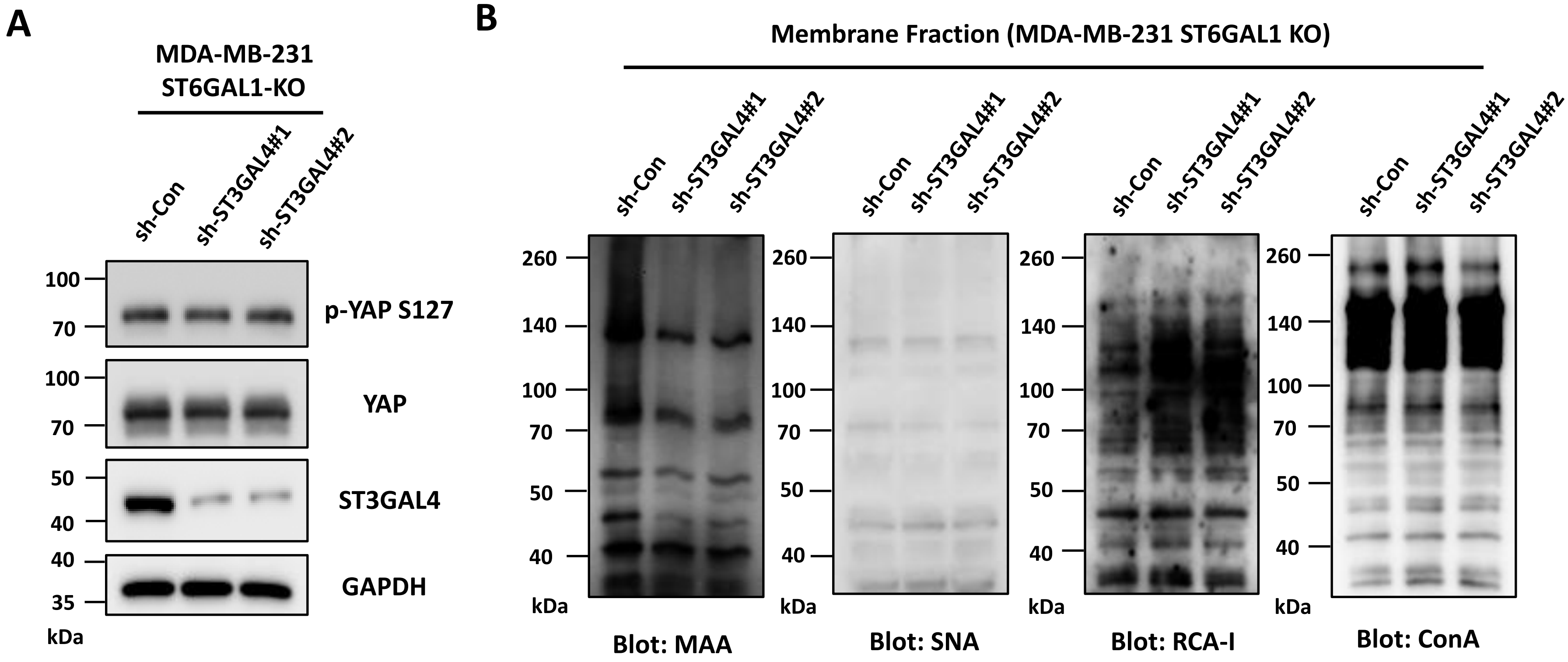

Supplementary Fig. 3

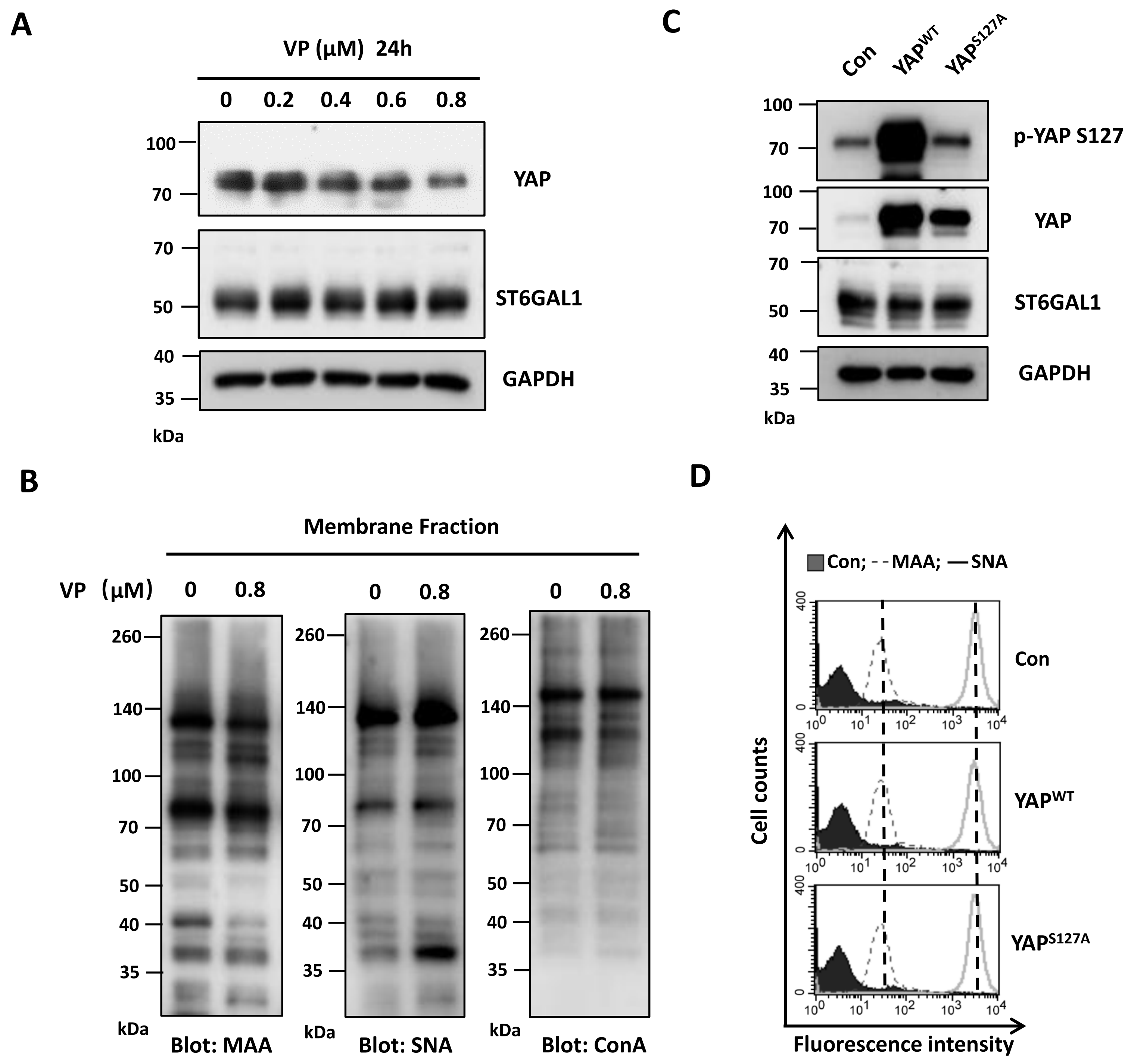

Supplementary Fig. 4

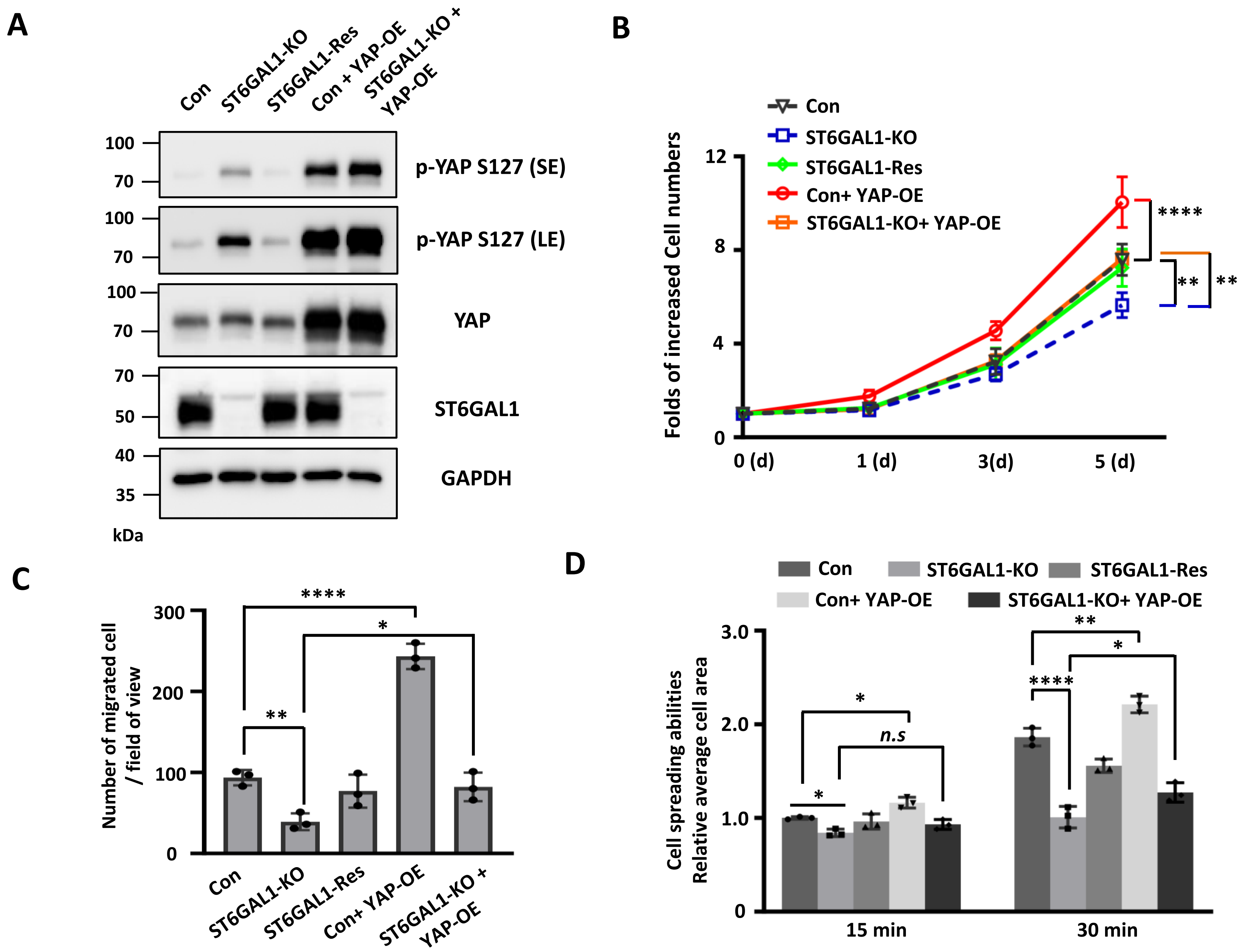

Supplementary Fig. 5

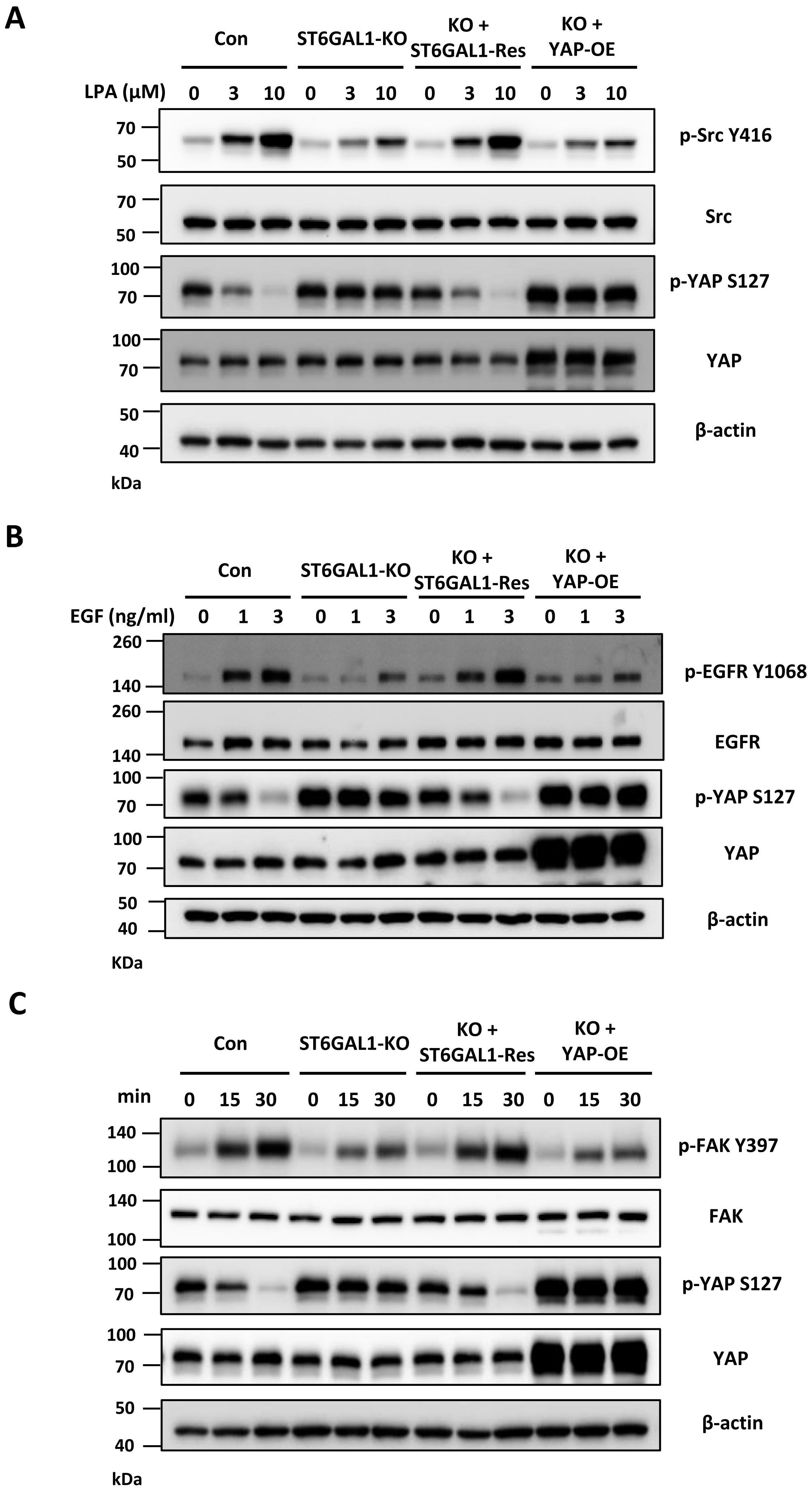

Supplementary Fig. 6

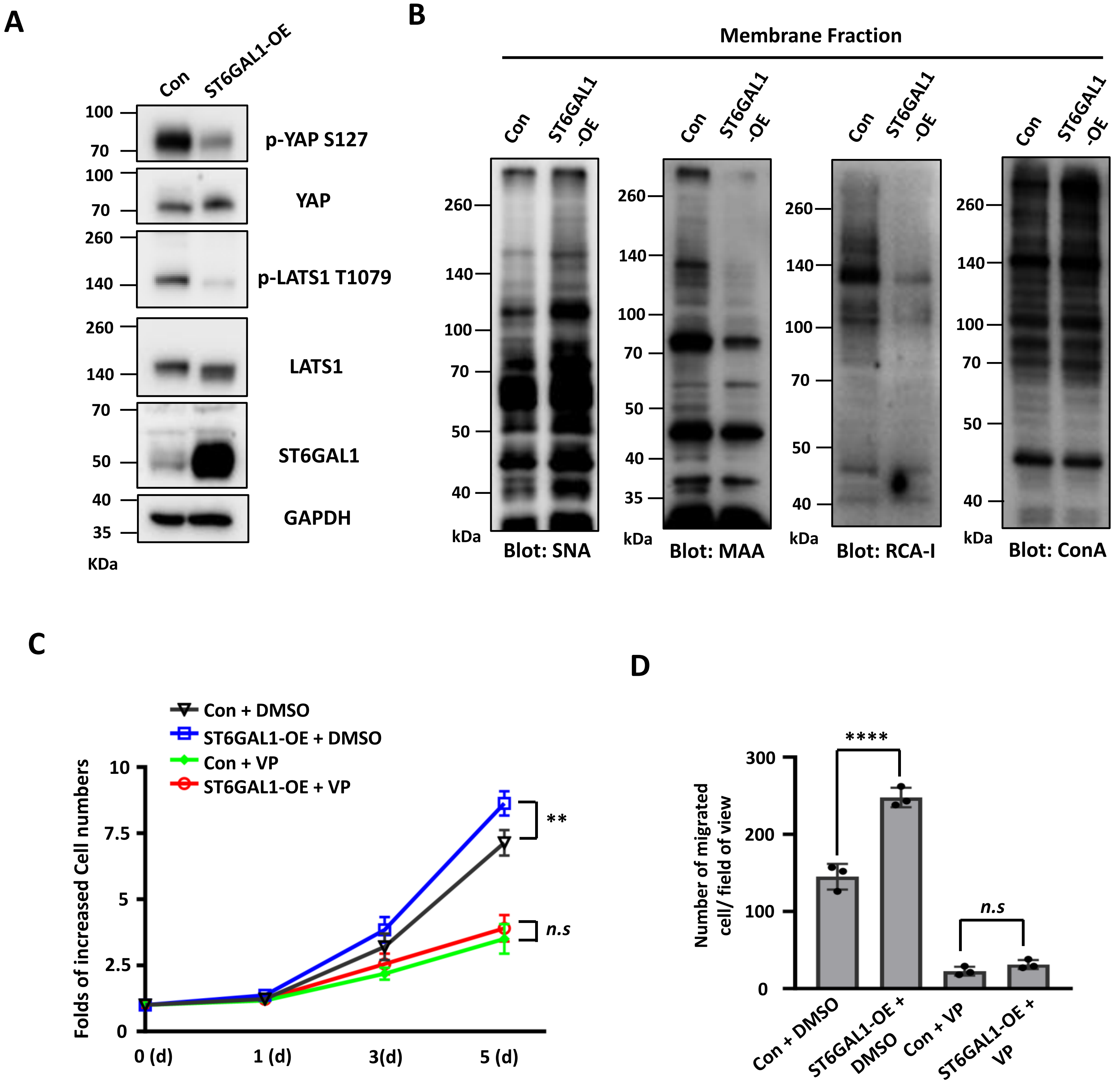

Supplement: Supplementary Figures [file mmc1.pdf]
