# Supplementary material for: Inhibitory effects of β-galactoside α2,6-sialyltransferase 1 on the Hippo pathway in breast cancer cells
Source: J Biol Chem. 2025 May 21;301(10):110266. doi: 10.1016/j.jbc.2025.110266 (PMC12550800; doi:10.1016/j.jbc.2025.110266)
Supplement: Supplementary Figure Legends [file mmc2.docx]

**Supplementary Figure legends**

**Fig. S1. Knockout of ST6GAL1 leads to functional inactivation of YAP in BT549 cells. (A)** Immunofluorescent staining of YAP protein in Con- and ST6GAL1-KO- BT549 cells. Nuclei were stained with TO-PRO-3. *Scale bar*, 20 μm. **(B)** The luciferase activity in Con-, ST6GAL1-KO-, and ST6GAL1-Res- BT549 cells. Cells were co-transfected with 8×GTIIC luciferase and TK-Renilla luciferase reporters; the reporter activities were measured using a Dual-Luciferase Reporter Assay System. **(C)** The mRNA expression of *ANKRD1*, *CTGF*, and *CYR61* genes in Con-, ST6GAL1-KO-, and ST6GAL1-Res- BT549 cells were detected by qPCR. Error bars in (B) and (C) are means±SD (*n* = 3 biological replicates, ***, *P* < 0.001, ****, *P* < 0.0001 are determined by one-way ANOVA with Tukey's *post hoc* test).

**Fig. S2. Effect of ST3GAL4 on YAP activation in ST6GAL1-KO-MDA-MB-231 cells.** **(A)** The cell lysates from Con-, ST3GAL4-sh#1-, and ST3GAL4-sh#2- ST6GAL1-KO-MDA-MB-231 cells were immunoblotted with anti-p-YAP S127, anti-YAP, anti-ST3GAL4, and anti-GAPDH antibodies. **(B)** The cell membrane fractions from the cells mentioned in (A) were blotted with ConA, RCA-I, MAA, and SNA lectins.

**Fig. S3. Effect of YAP protein levels on ST6GAL1, α2,3-, and α2,6-sialylation expression. (A)** MDA-MB-231 cells were pre-treated with different concentrations of Verteporfin (VP) for 24 h, and cell lysates were subjected to western blotting with anti-YAP, anti-ST6GAL1, and anti-GAPDH antibodies. **(B)** MDA-MB-231 cells were treated with or without 0.8 μM VP for 24 h, and the cell membrane fractions from indicated cells blotted with ConA, MAA, and SNA lectins. **(C)** The cell lysates from Con-, YAP-WT-overexpression (YAP^WT^)-, and YAP-S127A Mutant-overexpression (YAP^S127A^)-MDA-MB-231 stable cell lines were immunoblotted with anti-p-YAP S127, anti-YAP, anti-ST6GAL1, and anti-GAPDH antibodies. **(D)** The MDA-MB-231 derivative cell lines, as indicated in (C), were incubated with biotin-conjugated MAA (dotted line) or biotin-conjugated SNA (bold line) followed by incubation with appropriate Alexa Fluor 647 conjugate and then subjected to flow cytometry.

**Fig. S4. The ST6GAL1-YAP axis is involved in growth, migration, adhesion, and spreading of BT549 cells. (A)** The cell lysates from Con-, ST6GAL1-KO-, ST6GAL1-Res-, Con + YAP-OE-, and ST6GAL1-KO + YAP-OE- BT549 cells were immunoblotted with anti-p-YAP S127, anti-YAP, anti-ST6GAL1, and β-actin antibodies. LE, long exposure; SE, short exposure. **(B)** Growth curve of indicated BT549 cells mentioned in (A). **(C)** FN-mediated transwell migration assays of indicated BT549 cells mentioned in (A). **(D)** Cell spreading assay of indicated BT549 cells mentioned in (A). The error bars in (B-D) are presented as the mean±SD (*n* = 3 biological replicates, *n.s.* not statistically significant, *P* > 0.05; *, *P* < 0.05; **, *P* < 0.01; ****, *P* < 0.0001 are determined by two-way ANOVA with Tukey's *post hoc* test in panel B or one-way ANOVA with Tukey's *post hoc* test in panel C and D).

**Fig. S5. ST6GAL1 is involved in the LPA, EGF, and FN-mediated YAP activation responses in BT549 cells. (A and B)** After starvation for 24 h, the Con-, ST6GAL1-KO-, KO +ST6GAL1-Res-, and KO + YAP-OE- BT549 cells were treated with LPA for 30 min (A) or EGF for 20 min (B) at the indicated concentrations. WB analysis was performed with anti-p-Src Y416, anti-Src, anti-p-YAP S127, anti-YAP, and anti-β-actin antibodies in panel A and anti-p-EGFR Y1068, anti-EGFR, anti-p-YAP S127, anti-YAP, and anti-β-actin antibodies in panel B. **(C)** Indicated BT549 cells were detached, suspended in assay medium for 40 min, and then replated onto an FN-coated plate for the indicated times. WB analysis was performed with anti-p-FAK Y397, anti-FAK, anti-p-YAP S127, anti-YAP, and anti-β-actin antibodies.

**Fig. S6. ST6GAL1-YAP axis regulates BT549 cell growth and migration abilities. (A)** The cell lysates from Con- and ST6GAL1-OE- BT549 cells were immunoblotted with anti-p-YAP S127, anti-YAP, anti-p-LATS1 T1079, anti-LATS1, anti-ST6GAL1, and anti-GAPDH antibodies. **(B)** The cell membrane fractions from the indicated cells were blotted with ConA, RCA-I, MAA, and SNA lectins. **(C and D)** Comparison of cell growth (C) and migration (D) among Con-, ST6GAL1-OE- cells treated with DMSO or VP. The cell proliferation ability (C) and the migration ability toward FN (D) were detected. The error bars are presented as the mean±SD (*n* = 3 biological replicates, *n.s.* not statistically significant, *P* > 0.05; **, *P* < 0.01, ****, *P* < 0.0001 are determined by two-way ANOVA with Tukey's *post hoc* test in panel C or one-way ANOVA with Tukey's *post hoc* test in panel D).
